# CRISPRDetect: A flexible algorithm to define CRISPR arrays

**DOI:** 10.1186/s12864-016-2627-0

**Published:** 2016-05-17

**Authors:** Ambarish Biswas, Raymond H.J. Staals, Sergio E. Morales, Peter C. Fineran, Chris M. Brown

**Affiliations:** Department of Biochemistry, University of Otago, PO Box 56, Dunedin, 9054 New Zealand; Department of Microbiology and Immunology, University of Otago, PO Box 56, Dunedin, 9054 New Zealand; Genetics Otago, University of Otago, PO Box 56, Dunedin, 9054 New Zealand

**Keywords:** Phage resistance, Plasmids, Horizontal gene transfer, Cas, CRISPR, Small RNA targets, crRNA, Bioinformatics, Repeat elements

## Abstract

**Background:**

CRISPR (clustered regularly interspaced short palindromic repeats) RNAs provide the specificity for noncoding RNA-guided adaptive immune defence systems in prokaryotes. CRISPR arrays consist of repeat sequences separated by specific spacer sequences. CRISPR arrays have previously been identified in a large proportion of prokaryotic genomes. However, currently available detection algorithms do not utilise recently discovered features regarding CRISPR loci.

**Results:**

We have developed a new approach to automatically detect, predict and interactively refine CRISPR arrays. It is available as a web program and command line from bioanalysis.otago.ac.nz/CRISPRDetect. CRISPRDetect discovers putative arrays, extends the array by detecting additional variant repeats, corrects the direction of arrays, refines the repeat/spacer boundaries, and annotates different types of sequence variations (e.g. insertion/deletion) in near identical repeats. Due to these features, CRISPRDetect has significant advantages when compared to existing identification tools. As well as further support for small medium and large repeats, CRISPRDetect identified a class of arrays with ‘extra-large’ repeats in bacteria (repeats 44–50 nt). The CRISPRDetect output is integrated with other analysis tools. Notably, the predicted spacers can be directly utilised by CRISPRTarget to predict targets.

**Conclusion:**

CRISPRDetect enables more accurate detection of arrays and spacers and its gff output is suitable for inclusion in genome annotation pipelines and visualisation. It has been used to analyse all complete bacterial and archaeal reference genomes.

**Electronic supplementary material:**

The online version of this article (doi:10.1186/s12864-016-2627-0) contains supplementary material, which is available to authorized users.

## Background

CRISPR-Cas (clustered regularly interspaced short palindromic repeats-CRISPR associated) systems are adaptive immune systems in prokaryotes that provide protection from foreign genetic material, such as bacteriophages and plasmids. Specificity is provided by short noncoding RNAs (termed crRNAs; CRISPR RNAs) that recognise the invading DNA or RNA. These noncoding RNAs are derived from CRISPR arrays that possess near identical direct repeats, typically 21–48 bases long, punctuated by short non-identical ‘spacers’ that provide the immune ‘memory’ of these systems. [1–6]. CRISPR-Cas function requires a suite of Cas proteins encoded by *cas* genes, which are often located nearby the CRISPR loci (for reviews see [4–11]).

Analysis of CRISPR-Cas systems requires the detection of CRISPR arrays and their entire complement of spacer sequences. The computational recognition of CRISPRs has been approached in a number of different ways. Initially, CRISPRs were predicted by genomic pattern matching programs such as *PatScan* [12]. Then, to facilitate CRISPR prediction and analysis, a number of tools were developed, including both command-line executable programs (e.g. CRT [13], MINCED [14] and PILER-CR [15]) and web-applications (e.g. CRISPRFinder, CRISPI) [16, 17]. Recently, CRISPR prediction has been extended to metagenomic data [18–20].

The current prediction approaches have limitations, particularly in distinguishing CRISPRs from other types of repeats. In addition, many arrays show some mutation (substitutions or insertion and/or deletions), particularly at the 3’ end. Better approaches are needed to identify and represent these events. A drawback of the existing methods is that predictions do not fully utilise the available biological information. Current methods mainly rely on sequence similarities (and sometimes length distribution) in the repeats and spacers with predefined parameters, and do not search for key features of CRISPRs. For example, insertion, deletion and multiple point mutations may occur, then be propagated through subsequent repeats during duplication, or a portion or whole repeat and/or spacer could be deleted through recombination [21–26]. Furthermore, most of the existing programs fail to detect short or degenerate CRISPR arrays. Setting the parameters with high sensitivity may include these but will also lead to the identification of many non-CRISPR genomic repeats. Finding the true positives from such a large list of short CRISPR-like regions is laborious.

CRISPR arrays expand by duplication of the repeats and acquisition of spacers from the invading DNA [27]. This repeat duplication and spacer integration typically occurs at the leader end (AT-rich sequence containing the promoter) of the array [28, 29], although internal spacer acquisition can occur [30]. Repeats and spacers can also be lost by mutation, through small and large insertions or deletions, or recombination [21, 22, 26]. In addition, modelling has indicated there is a dynamic flux between acquisition and loss, driven by mutation and selection [31].

Most commonly used prediction tools do not assign strand or directionality to CRISPR arrays as part of the automated prediction process, resulting in roughly half of arrays being reported in the incorrect orientation. However, recent tools allow determination of CRISPR direction as a post-prediction step on arrays (CRISPRDirection), or repeat direction after array prediction (CRISPRstrand) [32, 33]. These developments have shown that the repeats can indicate the direction of CRISPR transcription [32–34]. For example, conserved sequence motifs (notably ATTGAAA(N)) at the 3’ of some repeats, are an indicator of the transcriptional direction [32, 33]. Therefore, it is important to accurately predict the repeat/spacer boundaries while predicting CRISPRs to correctly assign direction. In addition to sequence motifs, CRISPRDirection uses a range of predictive factors to determine array direction [32]. Defining direction is important to accurately identify spacers, since they are used to find their cognate DNA or RNA targets (termed protospacers) [35]. Since spacers are short (i.e. often ~30 nt), it is difficult to identify true targets and every additional correctly annotated nucleotide (nt) assists target detection. In Type I, Type II and Type V systems, the bases at one end of the spacer are usually part of a ‘seed’ sequence, that is critical for base-pairing, target recognition and interference [36–40]. Similarly, it is important to correctly identify the precise ends of the spacers to enable accurate prediction of important motifs flanking the protospacer, termed protospacer adjacent motifs (PAMs) [41]. PAMs are essential for target/non-target discrimination, so knowing their precise location is critical for identifying biologically relevant targets.

Towards the leader-distal (3’) end of CRISPR arrays, repeat mutations can accumulate. Furthermore, insertions and deletions can occur, especially in the 3’ end of CRISPR arrays [26, 42, 43]. These sequence deviations (repeat degeneracy and the presence of partially deleted repeats and/or spacers) mean that the 3’ ends of CRISPR arrays are often incorrectly detected. PILER-CR is currently the only program that handles insertions and/or deletions in repeats. The inability to detect these events means that we still have limited knowledge about how arrays degenerate to balance nascent spacer acquisitions at the leader end. The directional incorporation of new spacers implies a particular evolutionary history and can be used successfully in strain typing and evolutionary studies [44, 45]. Therefore, it would be informative if CRISPR detection provided a potential extension with lower repeat identity to test if degenerated, but still recognisable, repeats are present in the leader-distal end of the array.

Here, we developed CRISPRDetect, a web-based and command line tool, that enables accurate identification of CRISPR arrays in genomes, their direction, repeat spacer boundaries, substitutions, insertions or deletions in repeats and spacers and lists *cas* genes that are annotated in the genome. This data is combined into a searchable database, CRISPRBank, currently version 1.0. Spacer outputs from CRISPRDetect can then be directly used to search for targets in viral and other sequence databases using the linked tool, CRISPRTarget [35].

## Implementation

**Fig. 1:**
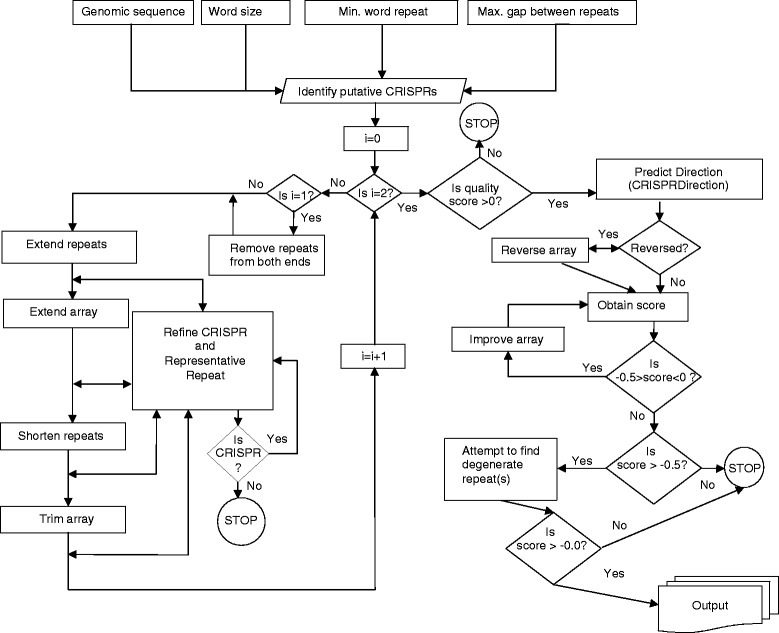
The CRISPRDetect automated pipeline. The modules that make up the pipeline are shown. In some cases there is an iterative repeat of processes, (iteration ‘0’ to i). See CRISPRDetect.pl for details. The interactive web implementation allows dynamic alteration of the parameters to suit the particular CRISPR array and genome

Figure 1 shows a schematic overview of the CRISPR identification and refinement process. Most existing tools identify CRISPRs using a default word length (e.g. 11) and minimum repetition (e.g. 3 or above). By default, CRISPRDetect searches with >2 repeats and a word length of ≥11 for a faster identification process, but it also allows identification of CRISPR arrays with only two repeats (i.e. 1 spacer) with a word size >5. CRISPRDetect uses five main processes to analyse a putative CRISPR: 1. repeat detection to give putative CRISPRs, 2. removal of CRISPR-like tandem repeats, 3. refinement, 4. determination of direction and similarity to characterised repeat families and 5. quality scoring.

### Detection of putative CRISPRs

CRISPRs are initially identified as two short stretches of identical sequences separated by a dissimilar short sequence. Since the shortest length of experimentally verified CRISPR repeats are about 23 nt [46], by default, we used a much shorter minimum word size of 11. However, CRISPRDetect can be run with word sizes >5. The minimum and maximum space (potential spacer length) between words are calculated using the following formulae.

$$ Minimum\  space\  between\  repeating\  words = 30\ \hbox{--}\ repeating- word- length $$

$$ Maximum\  space\  between\  repeating\  words=125 + repeating- word- length $$

The idea of not using fixed minimum and maximum lengths is to reduce user input as well as maintaining the speed. As the shortest verified repeat length is longer than 20 nt, this approach will ensure that CRISPRDetect will not miss any potential CRISPR. The default 11 nt word size ensures that potential shorter repeat (e.g. ~ 23 nt) with multiple base mismatches will be detected, while not compromising on speed for a typical bacterial or archaeal genome. This is done using regular expressions implemented in PERL.

### Removal of tandem repeats

The genomic regions containing the putative CRISPRs were analysed to identify repeats. Using the repeating word, the genomic regions are divided into sequence segments with every segment beginning with the repeated word. These repeated words are then aligned using ClustalW [47] and used to try and increase the initial repeat length of likely arrays as well as to eliminate simple tandem repeats. For the “spacers” of the putative CRISPRs that have <5 unaligned columns (i.e. are highly similar across the array), the putative CRISPR are marked as clear tandem repeats and discarded.

### Refinement of the putative CRISPR

CRISPRDetect supports eight independent refinement subroutines. These methods are used by default and applied in the specified order. Figure 1 shows the schematic diagram of the CRISPRDetect analysis pipeline, which is detailed in the following sections. However, each of these methods can be applied independently in an interactive manner to one or all CRISPRs using the CRISPRDetect web-server.

#### Extending the repeat end

**Fig. 2:**
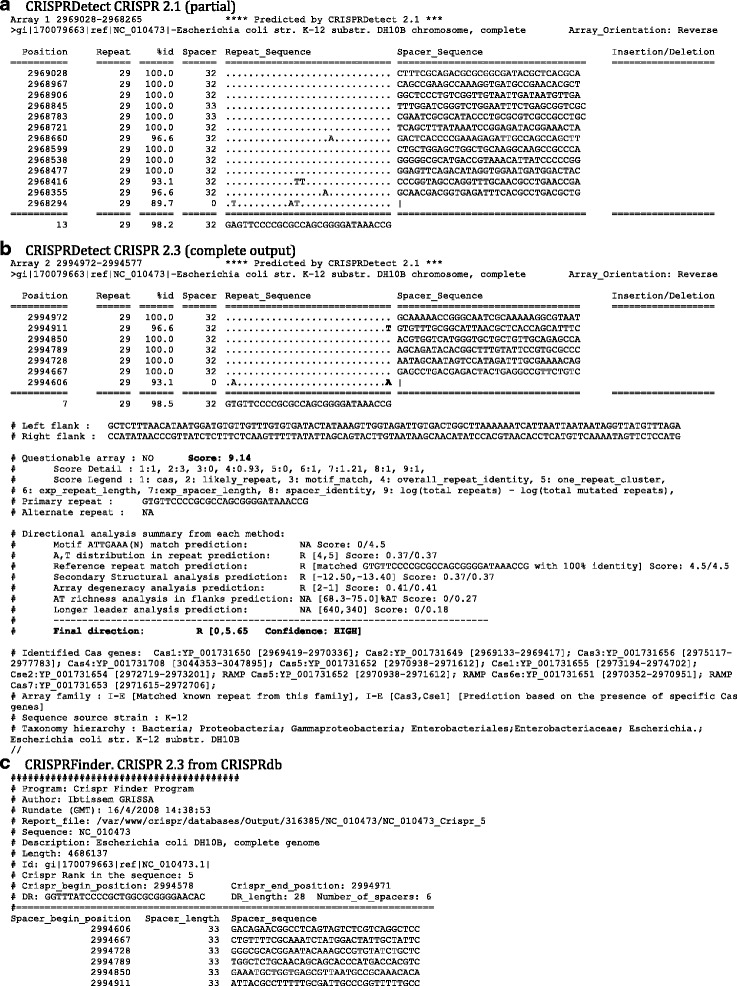
CRISPRDetect predictions for *E. coli* K-12*-* text output. CRISPRDetect identifies two CRISPR arrays in a K-12 genome, corresponding to the well characterised CRISPR 2.1 and 2.3 loci. This genome is provided as one of the test sets at http://bioanalysis.otago.ac.nz/CRISPRDetect/. CRISPRDetect output. *E. coli* arrays - both arrays are reverse-complemented in the CRISPRDetect prediction (based on matches to reference repeat and other features by CRISPRDirection) a CRISPR 2.1 The array section of the CRISPRDetect output is shown, showing base differences e.g. **a**. TT mutations in the repeat toward the predicted 3’ end. **b**. The full output is shown, and specific features are in bold. For CRISPR 2.3 the reference repeat match also permitted inclusion of the experimentally verified last base (G) in the repeat, although it varies in two of six repeats (the first and last, bold). The score is high (8.14) and the components are shown below. The Directional analysis gives a ‘HIGH’ confidence for the reverse orientation as shown. The *cas* genes identified in the ‘.gbk’ file are listed as are the signature genes for any family present (only I-E in this example). **c**. CRISPRFinder prediction for *E. coli* CRISPR 2.3 for comparison. Prediction obtained from CRISPRdb predicted by CRISPRFinder

Mutations at the ends of repeats may result in part of the repeat being included in the adjacent spacer sequences (e.g. Fig. 2C). CRISPRDetect progressively extends the repeat on both sides, comparing the bases from adjacent columns with minimum column identity by default of 75 % (range 0–100 %). Therefore for two or three repeats perfect identity is required, for four to seven one mismatch allowed, for eight two, and so forth.

Short repeats predicted initially may be bounded by a single column with low (e.g. 50 %) identity, but followed by columns with high identity. CRISPRDetect uses an adaptive method to extend the repeat if required, where instead of using only the primary column identity as a cutoff (default 75 %), it also uses an additional lower, ‘alternate column identity’ permitted for one column. The ‘alternate column identity’ is by default 50 % for arrays <7 repeats and 40 % for longer arrays. It is applied when a column has greater than the ‘alternative column identity’ but is followed by two or more columns with identity higher than the primary column identity (e.g. a column with only 4/10 identical bases, followed by two or more columns of 9/10. This has the added effect of extending the repeats of non-CRISPR tandem repeats split by low identity columns, this eliminates the ‘spacers' and identifies them as tandem repeats.

#### Selecting representative repeats

For most arrays there is very little dissimilarity among repeats and a representative repeat is easily selected. It is more difficult to identify a single representative repeat for shorter CRISPRs, those with frequent mutation in the repeats, or when more than one repeat sequence is found in longer arrays. The precise representative repeat is an important component of an array, as it helps to identify the family, direction, true spacer lengths, as well as the degenerated repeats at the end of array. This selection is repeated after every major operation on the array. CRISPRDetect selects the most common repeat as the ‘representative repeat’, with the next most common being the ‘alternative’ repeat.

#### Extend the array

This method progressively checks the flanking regions of the CRISPR arrays in windows within a distance equal to the length of the representative repeat plus 1.33 times the median spacer length for typical median spacer lengths (>15 and < 70, 2.5 × repeat length outside this range). The permitted minimum gap between newly identified repeats and existing repeats is 0 nucleotides to address total spacer loss, and the default upper limit is 125 nucleotides. The flanking region is compared with the Smith-Waterman algorithm (EMBOSS/water) with an increasing gap-penalty (starting from 5.5 to 10 in steps of 0.5) to identify the best non-gapped alignment. Once such an aligned region is identified, the region is extended either side accordingly, to match the representative repeat length. It is then further checked to ensure that the minimum repeat identity (default ≥67 %) is met (gaps, insertions and deletions are equally penalized with -1), and for all valid matches, a new repeat-spacer set is added to the array. This process is by default a dynamic one with the comparison being made to the adjacent repeat.

#### Refine the repeats

Initial repeat prediction may consist of additional bases at the ends that correctly belong to the spacers. This is due to situations where the first or last base of multiple spacers is nearly identical in an array. CRISPRDetect utilises a set of methods (comparison with a library of known repeats, known motifs (e.g. ATTGAAA(N)) found in the end of repeats, repeat end region degeneracy (default ≥20 % base mismatch)) to predict the correct repeat/spacer boundary. In the interactive mode, users can trim both sides of the repeats by any number of bases, as long as the repeat retains the minimum word length specified in the parameters for initial array prediction.

#### Trim the array - remove repeats that match poorly the representative repeat

Highly degenerated repeats can be falsely included after dynamically extending the CRISPRs, for example, if 2 repeats were added successively with 67 % identity the final repeat would have 45 % identity to the first. Repeats can be removed by requiring a minimum percentage identity between the representative repeat and terminal repeats. Trimming stops when a repeat has an identity above the cutoff (default >66 %) or the minimum number of repeats (default 3) specified is reached. This enables the user to have a simple means to remove sequences that are incorrectly assigned as degenerate repeats.

#### Correct gaps at repeat ends

After the initial repeat and spacer prediction, the repeat may contain terminal gaps or additional bases from the spacer, which can also make the spacer prediction incorrect. To refine the ends of the repeat, CRISPRDetect uses matching bases from the initially predicted spacer. For terminal insertions, the bases are labelled as insertions.

#### Representation of insertions in a small number of repeats of an array

**Fig. 3:**
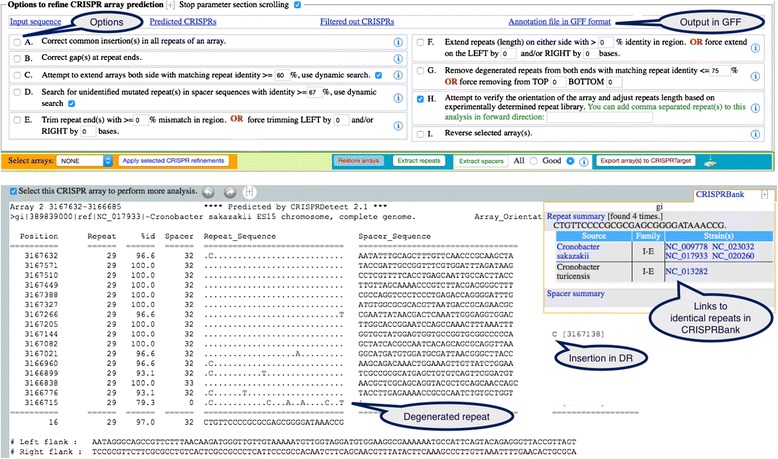
CRISPRDetect web output. An example of a predicted and automatically refined array from *Cronobacter sakazakii* ES15, which has 16 repeats, the last of which has degenerated. Options A-I are available for further interactive application of the selected processes to the selected array (Array 2 from this genome, array 1 is hidden). The array is shown in a standard format with substitutions in the repeat sequence shown. Insertions in one a repeat is indicated at the right. The quality score is high 8.87 (>4.0; max 13) and the score would be detailed in the next lines (as in Fig. 2, not shown). A link to CRISPRBank and initial analysis is shown in the top right and indicates that this exact repeat is found in five genomes (*Cronobacter* species). The annotation file in GFF can be downloaded for visualisation or further analysis (e.g. Fig. 6)

During alignment of the repeats, insertion of base(s) may have been identified. This results in introducing gap(s) in the visualisation of other repeats, including the representative repeat. To avoid these visual gaps in columns, CRISPRDetect denotes inserted bases as insertions in the array, which prevents the need to insert a gap character in the representative repeat (e.g. Fig. 3).

#### Identify mutated repeats in sequences initially predicted to be long spacers

When a substantial portion of a repeat and/or a repeat-spacer junction is deleted, the repeats fail to retain the minimum percentage identity and could be erroneously added to the next spacer, making these spacers appear unusually long. CRISPRDetect looks for such cases where the spacers are longer than the median spacer length with a user given minimum percentage identity between the representative repeat and the whole spacer, revealing not only partial repeat deletion, but also partial and/or total spacer deletion. These insertions and deletions are labeled in the output.

### Predicting direction

The direction of a CRISPR is predicted using the CRISPRDirection algorithm [32]. The arrays predicted in the reverse direction are automatically reverse complemented (i.e. they are displayed in the forward orientation, with the leader at the 5’ end). In the CRISPRDetect output, those that have been reversed are labelled accordingly.

### Predicting CRISPR-Cas Type

To give an indication of the CRISPR-Cas Type (e.g. Type I-E), two independent methods are used. Firstly when the representative repeat matches a known repeat that has been associated with a particular Type of CRISPR-Cas system (from a reference set [32]) the Type is indicated in the output. The reference set of validated repeats is also utilised in correcting repeat boundaries, scoring and validation of the arrays (later sections). Second if genomic annotation information is available (e.g. Genbank formatted files from Genbank/genomes), CRISPRDetect utilises the presence of annotated signature Cas genes (and synonyms) in the genome. The output lists all of the CRISPR-Cas Type(s) reported in the Genbank file.

### Scoring the quality of the predicted arrays

A scoring system gives each predicted array a score based on known biological properties. Each parameter has a positive or negative score and these are summed. These scores are detailed in Additional file 1: S1 and include: 1. the presence of annotated *cas1 or cas2* genes in a gbk or gbff file (+1, or 0); 2. a close match to known or confidently predicted repeats (+3); 3. specific sequence motifs at the 3’ end (+3); 4. a metric for identity within the repeats (+1); 5. a penalty for dissimilar repeats (-1.5); 6. metrics for the representative repeat length (-3 to +1); 7. metric for spacer length (0 to -3); 8. a penalty metric for identity among the spacers (-3 to +1) and 9. a penalty metric for dissimilarity among the repeats (-1 to +1). Each of these scores is listed in the output. A final score for each CRISPR array is determined by summing all the scores from the individual methods. The CRISPRs with negative scores are discarded, and the remaining CRISPRs are listed in order of position on the genome. Arrays with scores above 4.0 were classified as good quality based on comparison to the scores of arrays from experimentally validated species.

## Results and Discussion

### Overview

We aimed to develop a tool for improved detection of CRISPRs. CRISPRDetect was constructed to facilitate the identification and visualisation of the correct orientation of CRISPRs, spacer-repeat boundaries, substitution, insertion and deletion mutations, repeat similarity and the presence of *cas* genes in the genome. We define ‘true’ CRISPRs as experimentally determined arrays and ‘putative’ CRISPRs as those predicted computationally by CRISPRDetect or other methods. Putative CRISPRs are classified by CRISPRDetect as ‘good’, based on quality scoring criteria (≥4.0), or ‘Questionable’ (≥0 and <4.0) (Additional file S1). The most common repeat for each array is termed the representative repeat. The overall CRISPRDetect process is shown in Fig. 1.

CRISPRDetect was run on 2806 complete bacterial and archaeal genomes from GenBank/genomes (5262 sequences). This set of genomes was chosen to be comparable to that available for CRISPRFinder/CRISPRdb online (Feb 2016). Using the default settings, a total of 3901 CRISPRs were found, of these 3870 (97 %) were classified as ‘good’ arrays with a score of ≥4.0, repeats ≥3 and minimum repeat length ≥23. These arrays are further analysed here. There were 16,607 arrays flagged ‘Questionable’ with scores ≥ 0 and <4.0. Of these, 160 were further flagged as ‘Potential tandem repeats’.

CRISPRDetect modules performed iterative refinements on the arrays (see Implementation and specific examples below). Of the repeats in 3870 arrays, 12 % were not identical to the representative repeat, with 50 below 70 %, and 399 below 80 % identity. About half (as expected) were corrected in direction by CRISPRDetect and 1300 of these were corrected with high confidence (32). One hundred and sixty arrays were flagged as likely direct repeats (not having a repeat-dissimilar spacer structure) and are all ‘questionable’ arrays.

**Fig. 4:**
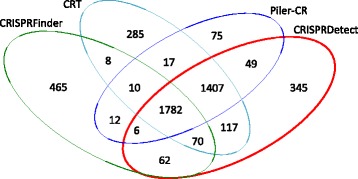
Comparison of the number of CRISPR arrays predicted by three existing methods compared with CRISPRDetect. Arrays with three or more repeats, and for CRISPRDetect a good quality score (>4.0) and ≥23 base repeat were counted

We compared these 3870 ‘good’ arrays to those predicted by three existing programs using their default parameters. A table of features in CRISPRDetect compared with CRT, PILER-CR and CRISPRFinder is presented in Additional file 1: Table S2. CRISPI was not tested as it is available online in an interactive mode only. CRT predicted 3681, PILER-CR 3743 and CRISPRFinder 2750 good CRISPR arrays (Fig. 4).

All programs predicted 1782 common arrays (Fig. 4). CRISPRDetect showed the highest concordance with PILER-CR and CRT (an additional 1407 arrays in common). Compared with the other methods CRISPRDetect predicted an additional 345 arrays. All arrays with scores >0 could be further analysed if desired (http://bioanalysis.otago.ac.nz/CRISPRBank/). Arrays can be selected for analysis by using a user selected cutoff score (e.g. 0.25, 3.0, or 5.0).

Arrays predicted using CRISPRDetect with similar settings to those used by CRISPRdb (CRISPRDetect score ≥4.0, repeat ≥3, min repeat length ≥23) were found in 75 % of archaeal genomes (124 of 165) and 45 % bacterial genomes (1179 of 2641). For CRISPRFinder/CRISPRdb the percentages of archaeal and bacterial genomes with predicted ‘convincing’ CRISPR structures are currently 83 % and 45 %.

Each of the other programs reported arrays that were not predicted when using the default settings for CRISPRDetect (Fig. 4). There were only 10 arrays predicted by the other three tools and not by CRISPRDetect. These arrays had between 3 to 5 repeats and all were predicted by CRISPRDetect, but had lower confidence scores. These arrays had scored lower, typically due to high similarity in spacers, or high numbers of mismatches in the repeats.

**Fig. 5:**
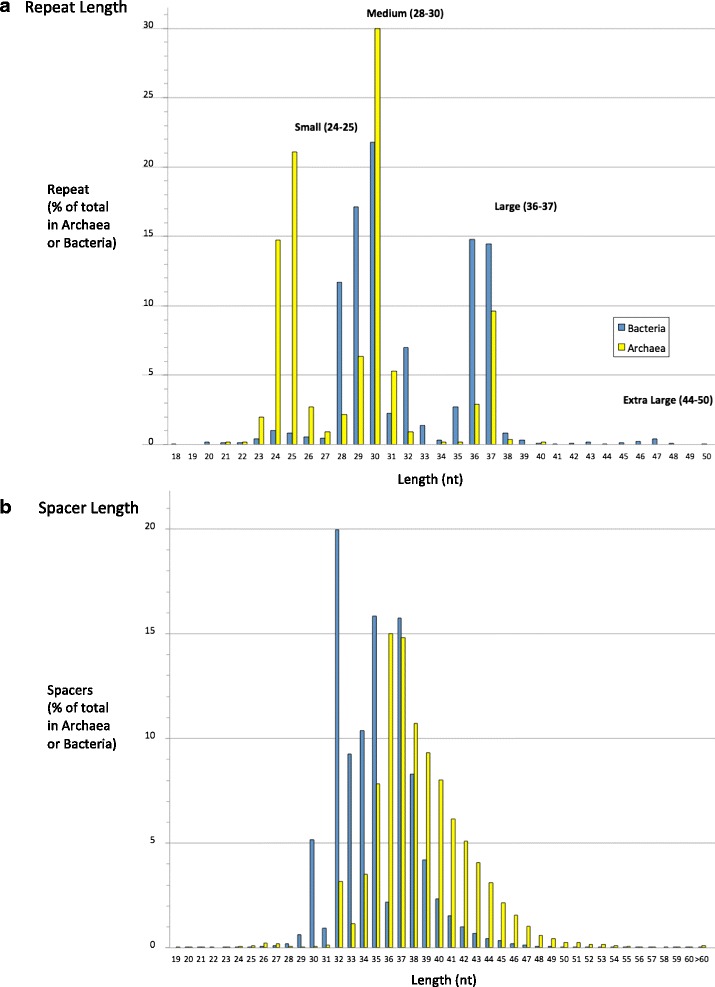
Sizes of CRISPR array repeats and spacers. **a** Distribution of sizes of the representative repeats for each array, the percentage of each length is shown separately for bacteria (blue) and archaea (yellow). Four size ranges- small, medium, large, and extra large are indicated. **b** Distribution of the median spacer size for each array. In (**a**) and (**b**) CRISPR arrays with ‘good’ scores (≥4.0) and three or more repeats from one strain for each species from Genbank/genomes were counted. For the same analysis including all strains, see Additional file 1: Figure S3

We used CRISPRDetect to determine the range of sizes of repeat and spacers (Fig. 5a-b). To minimize potential skew from overrepresented strains belonging to the same species in the databases, one strain from each species was analysed, and the length of the representative repeat and average spacer length determined. When compared with the same analysis performed on all arrays, there is no significant difference in the distribution (Additional file 1: Figure S3). The length of most repeats (96 %) are 24-37 nt and they can be classified into three major size ranges (small 24–25 nt, medium 28–30 nt, and large 36–37 nt) [46]. In contrast, there was a wide variation in spacer length across all genomes, but 97 % of the spacers are 29–43 nt (Fig. 5b). The most common spacer lengths are 32–37 nt in bacteria and 35–40 nt in archaea. These repeat classes are differently represented in archaea and bacteria. Small repeat (24–25 nt) are common in archaea (39.7 % of repeat) but not in bacteria (1.7 % of repeat). In bacteria, the large class is more common (25.8 % vs 11.5 %). Each range contains some repeat similar to experimentally determined CRISPR repeats. A new class including forty-four ‘extra large’ bacterial repeats (44–50 nt) is well supported by our predictions (Fig. 5a). This class was previously noted as associated with Type II-C proteins [48, 46]. Most are in the order *Flavobacteriaceae* within the Phylum *Bacteroidetes* and include *Capnocytophaga canimorsus* (NC_015846) 47 nt, 113 repeat; *Riemerella anatipestifer* species (e.g. NC_018609) 47 nt, 11–13 repeats; *Weeksella virosa* (NC_015144) 50 nt, 21 repeats. These arrays typically are adjacent to annotated *cas1*, *cas2* and *cas9* genes, and approximately half of these repeat have similar sequences at the 3’ end (UYACAAC). To see if prior analyses had omitted short repeats of genuine CRISPRs, we lowered the length restriction during detection. CRISPRDetect predicted 29 short repeats in bacteria and archaea with sizes <23 (the lower limit in CRISPRdb [46]). However, all but one are short arrays with typically less than 5 repeats, further experimental evidence would be required to determine if these are functional. Across all CRISPRs, the array with the greatest number of repeats is from the marine bacterium *Haliangium ochraceum* with 588 repeats of 36 bp (and two arrays nearby of 190 and 37 repeats with identical repeats).

It is possible for CRISPR arrays with only 1 ‘repeat’ and a portion of the leader to function for adaptation [27, 49, 50]. For common putative CRISPRs with only 2 repeats, they are flagged as ‘questionable’ by CRISPRFinder, and are not predicted by default by CRT or PILER-CR, as they would introduce many false positives. CRISPRDetect is able to discriminate between false positive and genuine CRISPR arrays by characterising the repeat and other scores. CRISPRDetect predicted an additional 770 arrays with just two repeats with score ≥1.5. Although none of these putative CRISPRs had a known reference repeat, 168 had the signature ATTGAA(N) sequence at the 3’ end so are likely new or divergent repeat sequences.

### Algorithms to refine the structures of arrays

The abovementioned benefits of using CRISPRDetect over other currently available software are nicely illustrated by analysis of *Escherichia coli* (NC_010473, 4.6 Mb). CRISPRDetect predicts two ‘good’ CRISPRs near 2.9 Mb on the genome (scores 7.90 and 8.14; maximum possible score of 13) (Fig. 2a–b). These arrays are well characterised experimentally [51, 52]. All previous programs made array predictions in the incorrect (reverse) orientation and inaccurately predict the repeat boundary for CRISPR2.3 (aka CRISPR II) by missing an incompletely conserved repeat base G on the 3’ end (Fig. 2c). This G has been experimentally shown to be an incompletely conserved part of the repeat [51]. CRISPRDetect automatically corrected the direction (using CRISPRDirection [32]) and the repeat boundaries (Fig. 2b). The boundaries were automatically corrected in the step where the representative repeats are compared to the library of known repeats (CRISPRBank, see Implementation section). The orientation and boundary corrections result in the precise spacer length and sequence identification, facilitating accurate subsequent analyses of protospacers, their target strand and their PAMs. Finally, identification of the likely CRISPR-Cas types (Type I-E in this case) was made by the presence of signature *cas* genes [7] in the annotated genome (Fig. 2b).

#### Insertion/deletion in repeats and spacers

Insertions, deletions and substitutions can occur in repeats and may be copied into new repeats during spacer acquisition [50]. CRISPRDetect detects repeat mutations, including insertions, deletions and substitutions. Of the existing tools, only PILER-CR represents substitutions in the repeat. In the cases of deletion (shorter repeats), the other tools usually incorrectly assign part of the spacer as part of the repeat in order to maintain the consensus repeat length. PILER-CR does not consistently predict the cases where the repeat-spacer junction has mutations within a few bases (<6) of the end of repeat. Furthermore, in PILER-CR, insertions are represented in one repeat, which creates a gap in all other repeats and the representative repeat. In CRISPRDetect, this is resolved with a new output notation (Fig. 2, and Fig. 3 CRISPRDetect online help). Insertions/deletions are listed to the right of the repeat-spacer unit, with their location denoted (e.g. C [3167138] means an insertion of the nucleotide C at position 3167138, Fig. 3; likely deletion of spacers is also denoted Additional file 1: Figure S4 and Figure S5). The deletion notation eliminates the need to artificially introduce gaps into multiple repeats, especially the representative repeat. In other cases, insertion of multiple bases towards the centre of a repeat may cause splitting the entire CRISPR into two or more short CRISPRs, which results in the inability to detect internal spacers. For example, in *Carboxydothermus hydrogenoformans*, a CRISPR array is split in two by PILER-CR (of 12 and 68 spacers), which CRISPRDetect corrects, leading to the identification of three extra spacers (83 spacers total, NC_007503-1949573-1944006). These CRISPR splitting events also complicate the analysis of leader regions, and the accurate assessment of the evolutionary history of acquisition events, since they would be analysed as two arrays rather than one. Partial deletions in spacers were detected by previous programs. However, these programs do not support the identification and visualization of complete spacer loss.

#### Identification of degenerated repeats in the spacer sequence

If repeats degenerate, they may not be recognised and can be included in subsequent spacers, resulting in erroneously long spacers. CRISPRDetect addresses this by searching for variant repeats with a lower identity threshold in all spacers with length 1.2-fold greater than the median spacer length in that array. If degenerate repeats are found, these are represented as repeats. For example in an atypical 8 repeat array from *Salmonella enterica* (Additional file 1: Figure S5), PILER-CR detects six repeats, with degenerated repeats being included as an unusually long 5th spacer (CRT and CRISPRFinder also make incorrect assignments, Additional file 1: Figure S5). CRISPRDetect predicts this array including 8 repeats (3 of the 8 repeats have small deletions at the repeat-spacer junction) with 5 typical spacers and 2 missing spacers at the 5’ end.

### Improving arrays by adjusting the repeat ends

CRISPRDetect uses a library of reference repeats (part of CRISPRBank) to automatically refine predicted repeat (Fig. 2a–b). This can be used in both an automatic and interactive way. If the new representative repeat matches a known reference repeat, then the repeat is extended or trimmed to have the reference length as described above for *E. coli* (Fig. 2a–b). In addition, if the representative repeat contains a known repeat boundary motif (e.g. ATTGAAA(N) 3’), then the 3’ end of the repeat is adjusted. This motif was found in 1070 arrays. Additionally, the web interface has the option to interactively increase or decrease the repeat length in an interactive array based on expert knowledge of the user.

### Identification of degenerated repeats and/or spacers beyond the end of an array

Repeats beyond the end of the array may degenerate by mutation and not be recognised. CRISPRDetect applies a lower match threshold to extend arrays. At the default settings this is set stringently, and arrays in the reference databank are predicted with this stringency (CRISPRBank). However, this is user-tuneable in both the automatic and interactive versions of the program. This allows users to investigate the decay of CRISPRs. Array extension is useful for analysing closely spaced arrays, separated by deleted or degenerate repeats or insertions. CRISPRDetect supports an extension, permitting repeat detection with identity as low as 35 % (Additional file 1: Figure S6, and Figure S7a, b). It also supports a ‘dynamic adaption’ method, where instead of using the global representative repeat, the nearest neighbouring repeat is used as a reference. One advantage of this method is that it allows dynamic adaptation where a repeat mutation has been propagated at one end of the array (Additional file 1: Figure S7c).

#### False positive predictions from tandem repeats

Other types of tandem repeats may be mis-identified as CRISPR arrays. No arrays with scores above 4 are flagged, there are 160 arrays with scores below 4 flagged as tandem repeats, (mean score 0.7). Additional file 1: Figure S8a provides an example of a predicted five repeat CRISPR (by CRT) with degenerated repeats being denoted as four spacers, CRISPRDetect does not predict this as an array. However, some likely arrays have a number of exactly identical spacers, followed by few non-identical spacers. For example, seven identical spacers are present in a 24 repeat array in *Methanocaldococcus jannaschii*, which is identified by CRISPRDetect (Additional file 1: Figure S8b and Figure S9).

#### Array orientation

Previous tools did not predict array orientation, until we developed CRISPRDirection, which corrects CRISPR orientation with ~94 % accuracy [32]. CRISPRDirection has a separate confidence score in the CRISPRDetect output (e.g. in *E. coli*, Additional file 1: Figure S4). An alternative would be to use CRISPRstrand [33], which predicts orientation using repeat but is not currently available as a command line program.

#### Internal database of CRISPRs (CRISPRBank)

As yet, there are no dynamically interactive CRISPR prediction tools to enable users to refine arrays. Although, CRISPRFinder and CRISPI are supported by some post-processing tools and a database (CRISPRdb), interaction between the prediction program and the database is not available. CRISPRDetect addresses this by incorporating a database of pre-computed CRISPRs (CRISPRBank) generated from all complete bacterial and archaeal genomes. Users can test newly predicted CRISPRs with a minimum score (default 4.0) during initial prediction, or once the output is generated. The representative repeat of each array can be directly searched in the CRISPRBank database, showing occurrences in other genomes. CRISPRBank currently contains 24,717 possible CRISPRs (score >0) with detailed information including family, direction and scores (the range of scores are shown in Additional file 1: Figure S10).

#### CRISPR-Cas Type indication

In CRISPRDetect and CRISPRBank predicted Types are indicated. This is based on the presence of signature *cas* genes (when annotated in the input Genbank format file) [7] and by similarity to repeat from known Types. In the output, CRISPRDetect lists the *cas* genes annotated, together with the sets of signature *cas* genes that were identified (Fig. 3). However, the lack of annotated *cas* genes in an output does not mean they are absent and further user analyses are advised. Analyses to find missing *cas* genes could include more sensitive searches for the *cas* genes, or use of the recently published compilation of *cas* genes [53] or CRISPRmap/CRISPRstrand analysis [33]. Proposed updates of the classification of CRISPR-Cas systems would be able to be incorporated into CRISPRDetect [48, 54, 55].

#### Scoring the quality of the arrays

The ‘quality’ of the final prediction is scored by a set of rules in CRISPRDetect. It scores each array with nine different CRISPR properties that includes both positive (e.g. length of repeat) and negative scores (e.g. a small penalty for the dissimilarity of the repeats) (Materials and Methods and Additional file 1). Arrays that score below a user given cutoff score are flagged as ‘questionable’. Arrays with scores <0 are not reported. These parameters are adjustable in both the automatic and interactive version. The presence of a known repeat gives an additional score (+3), therefore such repeats often have scores >6 (Additional file 1: Figure S10). However, many arrays score as good arrays (≥4) without a previously predicted repeat. The scores for all the predictions >0 from CRISPRDetect, and the scores for the arrays with experimentally confirmed repeats are shown in Additional file 1: Figure S10. CRISPRDetect defaults to a conservative score of 4.0, but lower values e.g. 3.0 could also be used for greater sensitivity (Additional file 1: Figure S10).

#### Direct link to CRISPRTarget for spacer analysis

From the CRISPRDetect output webpage, spacers can be sent directly to CRISPRTarget for target prediction in foreign DNA (e.g. the bacteriophage division of GenBank) [35]. CRISPRTarget uses a flexible algorithm that takes the formatted and predicted spacer sequences from CRISPRDetect (will also accept other formats) and uses these to search databases for targets.

#### Repeat analysis

CRISPRDetect shows any repeats that have an exact match in CRISPRBank. If desired, these repeat could be further analysed by CRISPRmap [33, 34]. CRISPRmap can classify the repeats based on sequence and structural similarity into one of 40 families or 33 structured motifs. This can then be used to predict the phylogenetic distribution of the family that the repeat matches.

#### Use in prokaryotic genome annotation pipelines

**Fig. 6:**
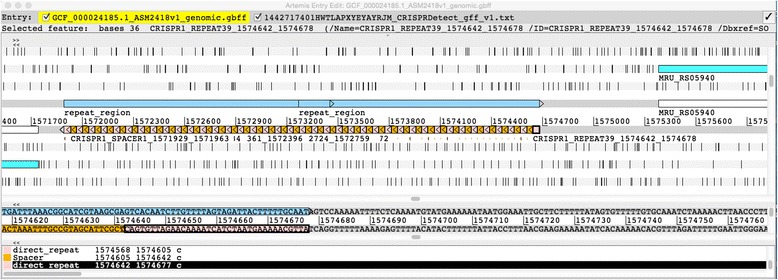
CRISPRDetect results on a genome browser. Genome feature format (gff) visualised in a genome browser (Artemis) [60]. This region has an array followed by an operon that includes some CRISPR associated genes. The figure shows a section of the RefSeq annotated version of *Methanobrevibacter ruminantium* genome [62]. The top line shows the annotation from the RefSeq file in GenBank (gbff) format. In the NCBI annotation pipeline the arrays are predicted by a combination of CRT and Piler-CR. These are annotated as a ‘repeat_region’s on the genome (light blue). The CRISPRDetect gff output file has been added to this annotation. Each repeat and spacer is shown in the indicated orientation

CRISPRDetect produces a gff output, which can be used for genome annotation or visualisation. Currently, CRISPR arrays may be annotated using a combination of modified CRT and PILER-CR (e.g. DOE-JGI Metagenome Annotation Pipeline v.4 [56, 57] and NCBI [58]). PROKKA also uses a modified version of CRT (MINCED) [14], whereas RAST uses Perl regular expressions to find repeat >24 [59]. Typically ncRNA predictions (e.g. CRISPR) are made then excluded from subsequent CDS prediction. CRISPRDetect could be incorporated into these pipelines in place of existing software using a high stringency (e.g. score >4) to avoid false positives and subsequent missing CDS predictions. For semi-automated finishing of genomes the gff output can be read into editors/viewers for example Artemis [60] or the Integrative Genomics Viewer (IGV) [61]. An array from the greenhouse gas producing archaea *Methanobrevibacter ruminantium* is shown in Fig. 6. The gff output is displayed alongside the RefSeq annotation of this genome. The fine structure of the array is shown in the likely orientation. The RefSeq pipeline annotation [58] broadly described as a ‘repeat-region’ is shown in light blue above.

## Conclusions

CRISPRDetect was designed to address limitations in current CRISPR prediction tools, and to include additional information that is now available. We focused on the prediction of CRISPR arrays by analysing both the CRISPR properties and distinguishing these from ‘CRISPR like’ repeats which can easily be predicted incorrectly as a CRISPR. CRISPRDetect, in combination with CRISPRBank and CRISPRTarget, now provides an integrated resource for the detection and analysis of CRISPRs (CRISPRSuite). We expect this suite will replace most existing CRISPR prediction tools.

The enhanced annotation of arrays reveals orientation, precise repeat-spacer boundaries, small and large mutations (substitution, deletion and insertions) in spacers and repeats, and additional features. This can be interrogated using a web interface, or be incorporated into genome annotation pipelines for improved gene annotation, where it would be included along with protein and other noncoding RNA predictions. We are now investigating these new features revealed by CRISPRDetect to generate further biological insight into CRISPR-Cas evolution and function.

### Availability of data and materials

Project name: CRISPRDetect

Project home page: http://bioanalysis.otago.ac.nz/CRISPRDetect/

Operating system(s): Platform independent

Programming language: PERL

Other requirements: Local installation- EMBOSS-water and seqret, RNAfold, clustalw, blastn, cd-hit-est

License: GNU GPL

Any restrictions to use by non-academics: no

## Additional file

Additional file 1: CRISPRDetect Additional files 1–10. (PDF 1633 kb)

## Abbreviations

CRISPR: clustered regularly interspaced short palindromic repeats
Cas: CRISPR associated
crRNA: CRISPR RNA
DR: direct repeat
PAM: protospacer adjacent motif
