# Supplementary material for: CRISPRDetect: A flexible algorithm to define CRISPR arrays
Source: BMC Genomics. 2016 May 17;17:356. doi: 10.1186/s12864-016-2627-0 (PMC4869251; doi:10.1186/s12864-016-2627-0)
Supplement: Additional file 1: — CRISPRDetect Additional files 1–10. (PDF 1633 kb) [file 12864_2016_2627_MOESM1_ESM.pdf]

Biswas et al 2016. Additional files

Additional File S1. CRISPRDetect array quality scoring scheme

1. Presence of either *cas1* or *cas2* genes in the genome is awarded (+1, or 0). (cas)
This method is only applied when an annotation file (NCBI gbk or gbff file) is used as input. The annotation files are searched (term based) to create a list of all *cas* genes present in the genome. The scoring system awards the quality score with '+1' when annotation of either *cas1* or *cas2* genes are present in the input file.

2. Match to known repeat using a set of reference repeats from high confidence arrays (+3). (**likely repeat)**
We use 26 experimentally verified representative repeats as reference and increased the set of known repeats by allowing up to 7 base mismatches. This extended set of ~400 repeat was used to predict a higher confidence set. Arrays were predicted then those with greater than 7 repeats and scores > 4 were used to predict a set of likely repeats. This file was converted in to a BLAST database and potential repeat searched against that with blastn-short which is optimised for short sequences. When a match is found, the array quality score is awarded '+3'.

3. Repeat has at least 23 bases and ATTGAAA(N) at the end (+3, or 0). (**motif_match)**
Another feature adapted from the CRISPRDirection algorithm is the presence of motif ATTGAAA(N) at the 3' end of repeats. We observed that, this motif is an accurate indicator of the direction of transcription. In that paper we also observed that all the potential repeats that are >=23nt long containing this motif were genuine CRISPRs. Hence, we used this information to contribute to the quality score, and the quality score is awarded with '+3' when the repeats are >=23nt long and contains ATTGAAA(N) at the 3' end.

4. Overall repeat identity within an array (0 to 1). (overall_repeat_identity)
The overall repeats identity score (S) is calculated using the following method

S= (average % identity of the repeats - 80)/20

The maximum possible positive score can be 1 (when all repeats are identical). However, the score will be negative, when the overall repeat identity is <80%.

5. The repeats in the array do not form one sequence similarity cluster (-1.5, or 0). (one_repeat_cluster)
The repeat are clustered using CD-HIT-EST if they form more than one cluster the quality score is penalized by '-1.5'.

6. Scoring the repeat lengths (range -3 to +1). (exp_repeat_length)
In this method, we use the table of repeat length distribution (Figure 3). The relative score (S) for a repeat of length (L) is determined using the following rules:

S= 0.25 + L/H [where, L>=23 and L =< 47;
H is the most abundant repeat length for bacteria or archaea]

S= -0.25*(23 - L) [where, L <23]

S= -0.25*(L - 47) [where, L >47]

The maximum negative score limit is set to -3, and maximum positive score limit is +1.

7. Scoring the spacer lengths (range -3 to +3). (exp_spacer_length)
In this method, each spacer of an array is independently scored, and counted towards a final spacer length score. The individual spacer length score (S) for a spacer with length (L) within the range 28-48 (see Fig 3B) are awarded a positive score using the formula:

S = 0.01 + N/H [where, 27< L =<48;
N= Total number of spacers of this length;
H= Most abundant spacer length for bacteria or archaea

Any spacer length outside this range is penalised by the following rule:

S=-0.10* (28 - L) [where, L<28]

S=-0.10* (L -48) [where, L>48]

Finally, an average spacer score for the current array is calculated using

Average score=Sum_of_scores/no_of_spacers

The maximum negative score limit is -3 and maximum positive score limit is +1.

8. Overall spacer identity (-3 to +1) (spacer_identity)
In this method we test the sequence (dis)similarity among all the spacers. If the spacers are all near identical it is more likely to be a direct repeat, possibly a tandem repeat rather than a CRISPR array. If the spacers belong to a total number of clusters (C) with identity >=80%, the spacer identity score (S) for an array with number of spacers (N) is calculated using the following rule:

S= -3 [where, C =< integer (N/2);]
S= 0.20*C [where, C > integer (N/2);]

The positive score limit is +1.

9. Scoring total number of identical repeats 0 to +1) (log(total repeats) - log(total mutated repeats))
Since longer arrays, and those with a greater number of identical repeats are more likely to be a true CRISPR, this scoring method uses both. If an array contains 'P' identical repeats out of the 'N' total number of repeats, then the score (S) is calculated using the following rule:

S= log (N) - log (N-P) [where, P=Identical repeats, N= total number of repeats]

The maximum positive score limit is +1.

In CRISPRDetect scoring system the sum total of the scores can range from +13 to -12.5.

Additional file S2 Comparison of three widely used CRISPR prediction tools with CRISPRDetect.

Feature	PILER-CR	CRT	CRISPI	CRISPRFinder	CRISPRDetect
Identifies insertion/deletions in repeats and spacers	yes	no	n/a	no	yes
Identifies complete spacer deletions	no	no	n/a	no	Yes
Identifies degenerate repeats in putative spacer sequence	no	no	no	no	yes
Identifies degenerated repeat and/or spacer in flanking regions	yes (threshold[1])	yes (threshold)	n/a	yes (uses dedicated function)	yes (uses dedicated function)
Identifies spacerless genomic tandem repeats	no	no	n/a	yes (with limitations)	yes
Extends arrays with a lower stringency. Joins closely spaced arrays separated by degenerated repeats	no	no	n/a	yes	yes
Removal of falsely predicted degenerated repeats from CRISPRs	n/a	n/a	n/a	no	yes
Shows flanking regions of the CRISPRs in the output	yes (partial, max 10nt)	no	no	no	yes
Identification of arrays with only 2 repeats	no	yes	n/a	yes	yes
Compares to a database of known repeats and features	no	no	yes	yes	yes
Annotates arrays at the end of circular genomes	no	no	n/a	no	yes

---

Determines family/type	no	no	no	no	yes
Determines the representative repeat	yes	no	no	yes	yes
Determines the CRISPR direction	no	no	no	no	yes
Shows *cas* genes present in the genome	no	no	yes	no	yes
Interactive web interface	no	no	yes	yes	yes
Pipeline version	yes	yes	no	yes (part)	yes
Supporting database	no	no	yes	yes (CRISPRdb)	yes (CRISPRBank)
User defined dictionary of spacers	no	no	no	yes (CRISPRtionary)	no
Compare flanks of two arrays	no	no	no	yes (FlankAlign)	**no**
Compare two more arrays	no	no	no	yes (CRISPRCompare)	**no**
Classify repeat into families	no	no	no	no	**yes**[2]

[1] In CRT 'threshold' refers to the global repeat/spacer identity parameter. PILER-CR and CRT do not support the use of specific parameters (e.g. a threshold) to identify degenerated repeat/spacers.
[2]. Gives an indication only, CRISPRMap gives a more comprehensive analysis.

Additional file S3.
As Figure 3 except that all strains are included.

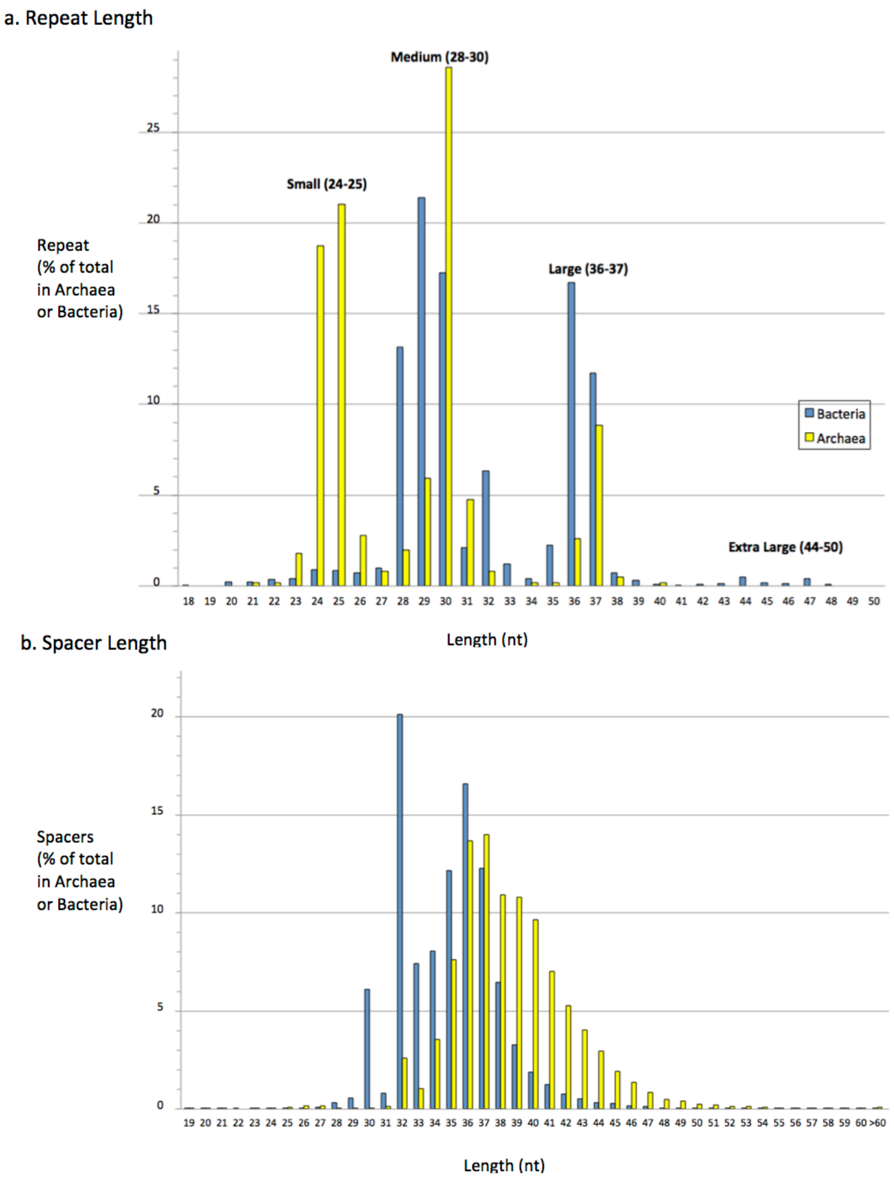

Additional File S4.

```
Initial Prediction

Repeat                          Spacer
=============================   ================================
..............-..............   GGACAGCAACCCGTGTCGGATATCAGACT
..............-..............   ACGCGAATCGCCAATCGCCGCCGCGTGAGC
GC...........-.........TGATCA   CGATGTATGCCGACCGTGATTTTTAGTCA
..............-..............   AGATACGCCTTTACGTCGCCTCTA
ATTC.........G...............   TAAAACACCGGTTGCGCAACCTGCAACGG
..............-..............   TCTAAATCTA -//-
=============================   ================================
CGGTTTATCCCCGC-TGGCGCGGGATCAC

Refined Prediction

Repeat                       Spacer                        Insertion/Deletion
============================ ============================= ==================
............................ GGACAGCAACCCGTGTCGGATATCAGACT
............................ ACGCGAATCGCCAATCGCCGCCGCGTGAG
............................ GATGTATGCCGACCGTGATTTTTAGTCA  C,T [X1,X2]
............................ AGATACGCCTTTACGTCGCCTCTAATTC
----........................ TAAAACACCGGTTGCGCAACC         G [X3], Deletion [X4]
............................ TCTAAATCTA -//-
============================ ============================= ==================
CGGTTTATCCCCGCTGGCGCGGGATCAC
```

S4A. A hypothetical array is shown before and after refinement. The bases belonging to the flanks are shown in green, repeat bases are shown in blue and insertion(s) are shown in red, X1 and X2 refers to corresponding positions of the insertions or deletions.

```
Initial prediction                             Refined Prediction
Repeat                 Spacer                  Repeat                 Spacer
====================== ====================    ====================== ====================
...................... GGACAGCAACCCGTGTCGGA    ...................... GGACAGCAACCCGTGTCGGA
...................... TCACACGCGAATCGCCAATC    ...................... TCACACGCGAATCGCCAATC
...................... GATGTATGCCGACCGTGATT    ...................... GATGTATGCCGACCGTGATT
...................... AGATACGCCTTTACGTCGCC    ...................... AGATACGCCTTTACGTCGCC
...................... GAAAACACCGGTTGCGCAAC    ...................... TAAAACACCGGTTGCGCAAC
...................... GCTAAAT -//-            ...................... TCTAAAT -//-
====================== ====================    ====================== ====================
GGTTTATTCCCCGCTATTGAAA                         GGTTTATTCCCCGCTACCGATA

GGTTTATTCCCCGCTATTGAAA-  Rep. repeat           GGTTTATTCCCCGCTACCGATA   Rep. repeat
               ATTGAAAN  Ref. Motif            -GTTTATTCCCCGCTACCGAT-   Ref. repeat

Repeat                  Spacer                 Repeat               Spacer
======================= ===================    ==================== ======================
....................... GACAGCAACCCGTGTCGGA    .................... AGGACAGCAACCCGTGTCGGAG
......................T CACACGCGAATCGCCAATC    .................... ATCACACGCGAATCGCCAATCG
....................... ATGTATGCCGACCGTGATT    .................... AGATGTATGCCGACCGTGATTG
......................A GATACGCCTTTACGTCGCC    .................... AAGATACGCCTTTACGTCGCCG
....................... AAAACACCGGTTGCGCAAC    .................... ATAAAACACCGGTTGCGCAACG
....................... CTAAAT -//-            .................... ATCTAAAT -//-
======================= ===================    ==================== ======================
GGTTTATTCCCCGCTATTGAAAG                        GTTTATTCCCCGCTACCGAT
```

S4B. Comparison to a reference motif and known repeat from CRISPRBank.

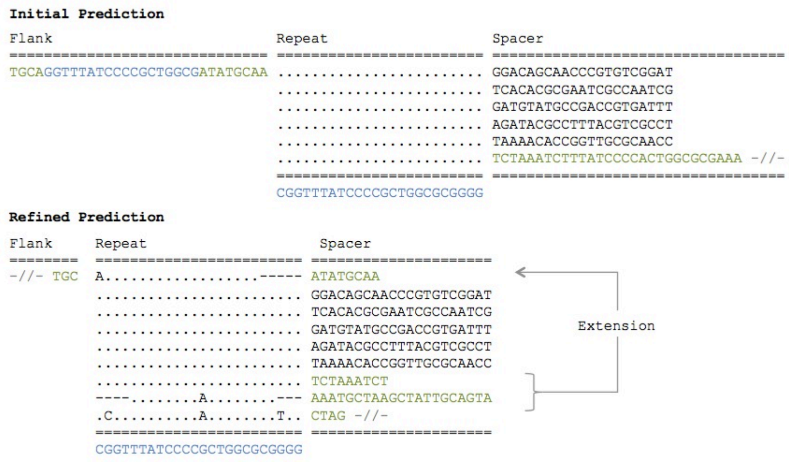

S4C. Extension of the array

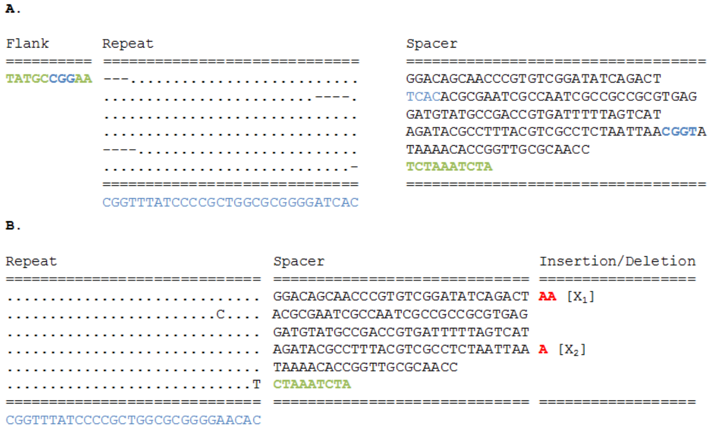

S4D. Inclusion of an initial predicted spacer bases in the repeats.

Additional File S5. Comparison of predictions from CRISPRDetect, PILER-CR, CRT and CRISPRFinder for the identification of partial/total spacer loss in *Salmonella enterica* subsp. *enterica* serovar Typhi str. CT18, array beginning at 2,926,568.

#------- predicted by CRISPRDetect

```
Array 1 2926568-2926181              **** Predicted by CRISPRDetect 2.1 ***
>gi|16758993|ref|NC_003198|-Salmonella enterica subsp. enterica serovar Typhi str. CT18,         Array_Orientation: Reverse

  Position     Repeat     %id  Spacer  Repeat_Sequence                 Spacer_Sequence                       Insertion/Deletion
==========     ====== ====== ====== ============================= ================================      ==================
   2926568         29   100.0      0 ............................. -                                     Deletion [2926539]
   2926539         26    86.2      0 ---A......................... -                                     Deletion [2926536]
   2926513         26    86.2     32 ---A......................... ATCCCCGCGGAGGTTGCGCAACCGGTGTTTTA
   2926455         29   100.0     32 ............................. CGCGCCAAAGAGGGCGACGTAAAGGCGTATCT
   2926394         29   100.0     32 ............................. GCGGTAAAAATCACGGTCGGCATACATCGTGG
   2926333         29   100.0     32 ............................. CAATTCACGCGGCGGCGATTGGCGATTCGCGT
   2926272         29   100.0     32 ............................. ATCTGTCTGATATCCGACACGGGTTGCTGTCC
   2926211         29   100.0      0 ............................. |                                     A [2926183]
==========     ====== ====== ====== ============================= ================================      ==================
         8         29    96.5     23 GTGTTCCCCGCGCCAGCGGGGATAAACCG

# Left flank :   TGTTGAAAATCAATAAGTTAGAGATCTTTAAAAATTAGGAAAAGTTGGTGGGTTTTTTGTGCGCTAAAAAAGTATTTAAATTCAATTGGGTAGATTTAGA
# Right flank :  TTTCACCAGCATATCAGGACGTTTTTTCCGCCTTCGCCAGCTCTTTTACCAACGGCAGCATTATCCGCACTACATCGCGGCTACGGCGCTCAATCCGCCC

# Questionable array : NO      Score: 7.77
#       Score Detail : 1:1, 2:3, 3:0, 4:0.83, 5:0, 6:1, 7:-0.06, 8:1, 9:1,
#
        Score Legend : 1: cas, 2: likely_repeat, 3: motif_match, 4: overall_repeat_identity, 5: one_repeat_cluster, 6: exp_repeat_length, 7:exp_s
pacer_length, 8: spacer_identity, 9: log(total repeats) - log(total mutated repeats),
# Primary repeat :     GTGTTCCCCGCGCCAGCGGGGATAAACCG
# Alternate repeat :   NA

# Directional analysis summary from each method:
#       Motif ATTGAAA(N) match prediction:         NA Score: 0/4.5
#       A,T distribution in repeat prediction:     R [4,5] Score: 0.37/0.37
#       Reference repeat match prediction:         R [matched GTGTTCCCCGCGCCAGCGGGGATAAACCG with 100% identity] Score: 4.5/4.5
#       Secondary Structural analysis prediction:  R [-12.50,-13.40] Score: 0.37/0.37
#       Array degeneracy analysis prediction:      R [1-0] Score: 0.41/0.41
#       AT richness analysis in flanks prediction: R [50.0-70.0]%AT Score: 0.27/0.27
#       Longer leader analysis prediction:         R [15,85] Score: 0.18/0.18
#       --------------------------------------------------------------------------
#       Final direction:         R [0,6.1   Confidence: HIGH]

# Identified Cas genes:  CRISPR/Cas system associated Cas1:NP_457327 [2926948-2927865]; CRISPR/Cas system associated Cse2:NP_457331 [2930385-
2930987]; CRISPR/Cas system associated RAMP super Unclassified_Cas_protein:NP_457328 [2927865-2928569]; Cas1:NP_457327 [2926948-
2927865]; Cas5:NP_457329 [2928569-2929294]; Cas7:NP_457330 [2929304-2930368]; Cse1:NP_457332 [2931004-2932539]; Cse2:NP_457331 [2930385-
```

```
2930987]; DinG helicase Csf4:NP_456328 [1840118-1842028]; RAMP Cas5:NP_457329 [2928569-2929294]; RAMP Cas6e:NP_457328 [2927865-
2928569]; Transcriptional regulator CasRa Unclassified_Cas_protein:NP_455939 [1470680-1471168];
# Array family : I-E [Matched known repeat from this family],
# Sequence source strain : CT18
# Taxonomy hierarchy : Bacteria; Proteobacteria; Gammaproteobacteria; Enterobacteriales;Enterobacteriaceae; Salmonella.; Salmonella enterica subs
p. enterica serovar Typhi str. CT18
//
```

#------ Predicted by PILER-CR

```
Array 1
>NC_003198

       Pos  Repeat     %id  Spacer  Left flank    Repeat                           Spacer
==========  ======  ======  ======  ==========    =============================    ======
   2926184      29    93.1      32  GGTGAAACGT    --...........................    GGACAGCAACCCGTGTCGGATATCAGACAGAT
   2926245      29   100.0      32  TCAGACAGAT    .............................    ACGCGAATCGCCAATCGCCGCCGCGTGAATTG
   2926306      29   100.0      32  GCGTGAATTG    .............................    CCACGATGTATGCCGACCGTGATTTTTACCGC
   2926367      29   100.0      32  TTTTTACCGC    .............................    AGATACGCCTTTACGTCGCCCTCTTTGGCGCG
   2926428      29   100.0      84  CTTTGGCGCG    .............................
TAAAACACCGGTTGCGCAACCTCCGCGGGGATCGGTTTATCCCCGCTGGCGCGGGGATCGGTTTATCCCCGCTGGCGCGGGGAT
   2926541      29   100.0          GCGCGGGGAT    .............................    TCTAAATCTA
==========  ======  ======  ======  ==========    =============================
         6      29              42                CGGTTTATCCCCGCTGGCGCGGGGAACAC
```

#------- predicted by CRT

```
CRISPR 10   Range: 2926182 - 2926567
POSITION        REPEAT                              SPACER
--------        -----------------------------       ------------------------------
2926182         GTGTTTATCCCCGCTGGCGCGGGGAACAC GGACAGCAACCCGTGTCGGATATCAGACAGAT      [ 29, 32 ]
2926243         CGGTTTATCCCCGCTGGCGCGGGGAACAC ACGCGAATCGCCAATCGCCGCCGCGTGAATTG      [ 29, 32 ]
2926304         CGGTTTATCCCCGCTGGCGCGGGGAACAC CCACGATGTATGCCGACCGTGATTTTTACCGC      [ 29, 32 ]
2926365         CGGTTTATCCCCGCTGGCGCGGGGAACAC AGATACGCCTTTACGTCGCCCTCTTTGGCGCG      [ 29, 32 ]
2926426         CGGTTTATCCCCGCTGGCGCGGGGAACAC TAAAACACCGGTTGCGCAACCTCCGCGGGGAT      [ 29, 32 ]
2926487         CGGTTTATCCCCGCTGGCGCGGGGATCGG TTTATCCCCGCTGGCGCGGGGAT       [ 29, 23 ]
2926539         CGGTTTATCCCCGCTGGCGCGGGGAACAC
--------        -----------------------------       ------------------------------
Repeats: 7      Average Length: 29                  Average Length: 30
```

#------ predicted by CRISPRFinder

```
########################################
# Program: Crispr Finder Program
# Author: Ibtissem GRISSA
# Rundate (GMT): 16/11/2006 15:27:24
# Report_file: /var/www/crispr/databases/Output/220341/NC_003198/NC_003198_1
########################################
```

```
#=======================================
#
# Sequence: NC_003198
# Description: Salmonella enterica subsp. enterica serovar Typhi str. CT18, complete genome
# Length: 4809037
# Id: gi|16758993|ref|NC_003198.1|
#
#=========================================================================
# Crispr Rank in the sequence: 1
# Crispr_begin_position: 2926182      Crispr_end_position: 2926515
# DR: CGGTTTATCCCCGCTGGCGCGGGGAACAC    DR_length: 29  Number_of_spacers: 4
Spacer_begin_position   Spacer_length  Spacer_sequence
              2926211             32  GGACAGCAACCCGTGTCGGATATCAGACAGAT

              2926272             32  ACGCGAATCGCCAATCGCCGCCGCGTGAATTG

              2926333             32  CCACGATGTATGCCGACCGTGATTTTTACCGC

              2926394             32  AGATACGCCTTTACGTCGCCCTCTTTGGCGCG

              2926455             32  TAAAACACCGGTTGCGCAACCTCCGCGGGGAT

#=========================================================================
########################################
```

Additional File S6. Reduction in the repeat identity in *E. coli* K-12 DH10B chromosome (array beginning at 2,969,028) identifies additional repeat and spacer in CRISPRDetect web application.

#------ CRISPRDetect initial output

```
Array 1 2969028-2968265              **** Predicted by CRISPRDetect 2.1 ***
>gi|170079663|ref|NC_010473|-Escherichia coli str. K-12 substr. DH10B chromosome, complete              Array_Orientation: Reverse

  Position      Repeat     %id  Spacer  Repeat_Sequence              Spacer_Sequence                       Insertion/Deletion
==========      ======  ======  ======  ============================= =================================    ==================
   2969028          29   100.0      32  ............................. CTTTCGCAGACGCGCGGCGATACGCTCACGCA
   2968967          29   100.0      32  ............................. CAGCCGAAGCCAAAGGTGATGCCGAACACGCT
   2968906          29   100.0      32  ............................. GGCTCCCTGTCGGTTGTAATTGATAATGTTGA
   2968845          29   100.0      33  ............................. TTTGGATCGGGTCTGGAATTTCTGAGCGGTCGC
   2968783          29   100.0      33  ............................. CGAATCGCGCATACCCTGCGCGTCGCCGCCTGC
   2968721          29   100.0      32  ............................. TCAGCTTTATAAATCCGGAGATACGGAAACTA
   2968660          29    96.6      32  ..................A.......... GACTCACCCCGAAAGAGATTGCCAGCCAGCTT
   2968599          29   100.0      32  ............................. CTGCTGGAGCTGGCTGCAAGGCAAGCCGCCCA
   2968538          29   100.0      32  ............................. GGGGGCGCATGACCGTAAACATTATCCCCCGG
```

```
   2968477         29  100.0      32  ............................. GGAGTTCAGACATAGGTGGAATGATGGACTAC
   2968416         29   93.1      32  ............TT............... CCCGGTAGCCAGGTTTGCAACGCCTGAACCGA
   2968355         29   96.6      32  .................A........... GCAACGACGGTGAGATTTCACGCCTGACGCTG
   2968294         29   89.7       0  .T.........AT................ |
==========     ====== ======  ======  ============================= =================================     ==================
        13         29   98.2      32  GAGTTCCCCGCGCCAGCGGGGATAAACCG

# Left flank :   AAGAATTAGCTGATCTTTAATAATAAGGAAATGTTACATTAAGGTTGGTGGGTTGTTTTTATGGGAAAAAATGCTTTAAGAACAAATGTATACTTTTAGA
# Right flank :  GGCGCACTGGATGCGATGATGGATATCACTTGGAGTTCCCCCGCCTCTGCGGTAGAACTCCCAGCTCCCATTTTCAAACCCATCAAGACGCCTTCGCCAA
```

#--- CRISPRDetect output (partial output) [after reducing the repeat identity to 55% from the default 80%, and using dynamic search]

```
Array 3 2969028-2968205        **** Predicted by CRISPRDetect 2.1 ***
>NC_010473|Escherichia-coli str. K-12 substr. DH10B chromosome, complete    Array_Orientation: Reverse

  Position     Repeat     %id  Spacer  Repeat_Sequence               Spacer_Sequence                       Insertion/Deletion
==========     ====== ======  ======  ============================= =================================     ==================
   2969028         29  100.0      32  ............................. CTTTCGCAGACGCGCGGCGATACGCTCACGCA
   2968967         29  100.0      32  ............................. CAGCCGAAGCCAAAGGTGATGCCGAACACGCT
   2968906         29  100.0      32  ............................. GGCTCCCTGTCGGTTGTAATTGATAATGTTGA
   2968845         29  100.0      33  ............................. TTTGGATCGGGTCTGGAATTTCTGAGCGGTCGC
   2968783         29  100.0      33  ............................. CGAATCGCGCATACCCTGCGCGTCGCCGCCTGC
   2968721         29  100.0      32  ............................. TCAGCTTTATAAATCCGGAGATACGGAAACTA
   2968660         29   96.6      32  ..................A.......... GACTCACCCCGAAAGAGATTGCCAGCCAGCTT
   2968599         29  100.0      32  ............................. CTGCTGGAGCTGGCTGCAAGGCAAGCCGCCCA
   2968538         29  100.0      32  ............................. GGGGGCGCATGACCGTAAACATTATCCCCCGG
   2968477         29  100.0      32  ............................. GGAGTTCAGACATAGGTGGAATGATGGACTAC
   2968416         29   93.1      32  ............TT............... CCCGGTAGCCAGGTTTGCAACGCCTGAACCGA
   2968355         29   96.6      32  .................A........... GCAACGACGGTGAGATTTCACGCCTGACGCTG
   2968294         29   89.7      32  .T.........AT................ GGCGCACTGGATGCGATGATGGATATCACTTG
   2968233         28   69.0       0  ...........CT.T....T-.G..CT.C |                                     C [2968220]
==========     ====== ======  ======  ============================= =================================     ==================
        14         29   96.1      32  GAGTTCCCCGCGCCAGCGGGGATAAACCG
```

Additional File S7. A. An example of array extension in *Leptospira interrogans* serovar Lai str. 56601 chromosome I, array beginning at 3,163,253.

#---------- predicted by PILER-CR

```
Array 1
>NC_004342

       Pos  Repeat     %id  Spacer  Left flank    Repeat                                     Spacer
==========  ======  ======  ======  ==========    =======================================    ======
   3163253      39    97.4      32  AATAAAATGC    .....................................C.    GTTCTGATTTTTTCTTTTCCTTCCTTTTGTTA
   3163324      39   100.0      32  CCTTTTGTTA    .......................................    CCCACGATACTACCTGTCAGACCGTGCCCGGA
   3163395      39    97.4          CGTGCCCGGA    .....................................G.    ACTCCTCGA
==========  ======  ======  ======  ==========    =======================================
         3      39              32                TCTGAATATAACTTTGATGCCGTTAGGCGTTGAGCACAC
```

#---------- predicted by CRISPRDetect (partial output)

```
Array 2 3163253-3163504              **** Predicted by CRISPRDetect 2.1 ***
>gi|294827553|ref|NC_004342|-Leptospira interrogans serovar Lai str. 56601 chromosome I,         Array_Orientation: Forward

  Position     Repeat     %id  Spacer  Repeat_Sequence                       Spacer_Sequence                      Insertion/Deletion
==========     ======  ======  ======  ===================================== ==================================   ==================
   3163253         37   100.0      34  ..................................... CCGTTCTGATTTTTTCTTTTCCTTCCTTTTGTTA
   3163324         37   100.0      34  ..................................... ACCCCACGATACTACCTGTCAGACCGTGCCCGGA
   3163395         37   100.0      34  ..................................... GCACTCCTCGAACTGGTAAAACTACCGATGCTCG
   3163466         37    89.2       0  C..............................T..A.T |                                    G [3163493]
==========     ======  ======  ======  ===================================== ==================================   ==================
         4         37    97.3      34  TCTGAATATAACTTTGATGCCGTTAGGCGTTGAGCAC

# Left flank :   AACATGAAAAATAACGATAAAAAACGATATACTTGTTCTCCTCCTTTGAAAACACTCATATCCACAATTTATGCTTATAAAAGCCATTTAAATAAAATGC
# Right flank :  GAAGAGAAGATTTGTTTTGGCCCAAATTGTTTCGCACCAAACGTGCAAACGGATAAAATGTAGGAACTACTACTTTTTCGAAAAACAGTACTTTGTTCAA

# Questionable array : NO      Score: 5.14
#       Score Detail : 1:1, 2:0, 3:0, 4:0.86, 5:0, 6:1, 7:0.68, 8:0.6, 9:1,
#
       Score Legend : 1: cas, 2: likely_repeat, 3: motif_match, 4: overall_repeat_identity, 5: one_repeat_cluster, 6: exp_repeat_length, 7:exp_s
pacer_length, 8: spacer_identity, 9: log(total repeats) - log(total mutated repeats),
# Primary repeat :     TCTGAATATAACTTTGATGCCGTTAGGCGTTGAGCAC
# Alternate repeat :   NA

# Directional analysis summary from each method:
#       Motif ATTGAAA(N) match prediction:         NA Score: 0/4.5
#       A,T distribution in repeat prediction:     NA [Repeat is AT rich:56.76%AT]
#       Reference repeat match prediction:         NA
#       Secondary Structural analysis prediction:  F [-7.60,-3.60] Score: 0.37/0.37
#       Array degeneracy analysis prediction:      F [0-5] Score: 0.41/0.41
#       AT richness analysis in flanks prediction: F [71.7-61.7]%AT Score: 0.27/0.27
#       Longer leader analysis prediction:         R [366,995] Score: 0.18/0.18
#       --------------------------------------------------------------------------
#       Final direction:         F [1.05,0.18   Confidence: HIGH]
```

S7B Array extension by joining closely spaced arrays in *Myxococcus fulvus* HW-1 chromosome, array beginning at 2,683,776.
PILER-CR predicted two CRISPRs with the same representative repeat 'GTCGCTCCCCGTGAACGCGGGGAGCGTGGGTTGAAAC' separated by 520 bases. Since PILER-CR and CRT do not support providing any specific gap parameter to join closely spaced array, no further analysis was possible. Providing a higher maximum spacer length of 520 had no effect on the outcome. More detailed analysis using

CRISPRDetect revealed additional repeats and spacers within this 520 base long region. Using the program defaults, both CRISPRDetect and CRISPRFinder, successfully identified a longer CRISPR array instead of two shorter one. However, the array predicted by CRISPRFinder only had 80 repeats, whereas the array predicted by CRISPRDetect contained 104 repeats.

#---- predicted by PILER-CR

```
Array 9
>NC_015711

       Pos  Repeat     %id  Spacer  Left flank    Repeat                                   Spacer
==========  ======  ======  ======  ==========    =====================================    ======
   2683776      37   100.0      36  CGCGGAAATC    .....................................    CTTCCACCACTGCCAATGCGCCGCTGGGCACCATTC
   2683849      37    97.3      34  GGCACCATTC    ........T............................    GCGTCGGCCTCCAGCGAGTCGGGCAGCAGGAGAA
   2683920      37   100.0      32  AGCAGGAGAA    .....................................    GCCATGCAGTGGCTGGAGGAGTTCGTCCTGCC
   2683989      37   100.0      34  TCGTCCTGCC    .....................................    CCTCGCCACCCTCCGCCAAATTTGCCACCGCGTG
   2684060      37   100.0      32  CCACCGCGTG    .....................................    ACCGTGGACGGCCGCAACTGGCTGCCCTGCAC
   2684129      37   100.0      35  TGCCCTGCAC    .....................................    AAGTCTTCCGTGTTCTCCATGTCGCTTCCCGCCTG
   2684201      37   100.0      37  TTCCCGCCTG    .....................................    CCATGACGCTGCCCCTCTCGCGGGCCTCGAGCCCGAT
   2684275      37   100.0      35  CGAGCCCGAT    .....................................    ACCACCTTGTCGCTTTGGTGGTCGGCGTAGTGGAT
   2684347      37   100.0      32  CGTAGTGGAT    .....................................    GTCACCCACAGCCTGCCCAGCGGCGCCACCTG
   2684416      37   100.0      33  GCGCCACCTG    .....................................    ACCCTGGAGGTCGTAGGTCGAACTTCAAGCCGA
   2684486      37   100.0      34  TTCAAGCCGA    .....................................    AAGGCGACCGTCCGCGCGCTGTCCACCACGGCCT
   2684557      37   100.0      35  ACCACGGCCT    .....................................    CCTCCTCCTGGAGGGCGGGGTGAATAGCCTGCGCG
   2684629      37   100.0      33  AGCCTGCGCG    .....................................    TTCGAACTGCGCGACCGGCGCGTGCAGTTCACC
   2684699      37   100.0          GCAGTTCACC    .....................................    TGGCACTGCC
==========  ======  ======  ======  ==========    =====================================
        14      37              34                GTCGCTCCCCGTGAACGCGGGGAGCGTGGGTTGAAAC


Array 10
>NC_015711

       Pos  Repeat     %id  Spacer  Left flank    Repeat                                   Spacer
==========  ======  ======  ======  ==========    =====================================    ======
   2685256      37   100.0      35  ACCACGGCCT    .....................................    CCTCCTCCTGGAGGGCGGGGTGAATAGCCTGCGCG
   2685328      37   100.0      33  AGCCTGCGCG    .....................................    TTCGAACTGCGCGACCGGCGCGTGCAGTTCACC
   2685398      37   100.0      35  GCAGTTCACC    .....................................    TGGCACTGCCGCTTGACCTCCGCAGGCTTGAAGGT
   2685470      37   100.0      33  GCTTGAAGGT    .....................................    CCCTGACGCTGCCCCTCTCGCGGGCCTCGGGCC
   2685540      37   100.0      34  GCCTCGGGCC    .....................................    AGCGGGCCGAACATCGCCCGGCTGCCGGGCGCTT
   2685611      37   100.0      32  CCGGGCGCTT    .....................................    ACCGTCACCGAGGTTATCCCACCGCTCCCCAC
   2685680      37   100.0      34  CGCTCCCCAC    .....................................    GCGGGAATCATTACCGACCCGCATGGTGTCCCGG
   2685751      37   100.0      32  GGTGTCCCGG    .....................................    GCAACATCCAGGTGAAGCCCGGCGCGGAAATC
   2685820      37   100.0      34  CGCGGAAATC    .....................................    AAGATGACGACACCGCAGCGGGCGCACGTCTGCT
   2685891      37   100.0      36  CACGTCTGCT    .....................................    CTTCCACCACTGCCAATGCGCCGCTGGGCACCATTC
   2685964      37    97.3      34  GGCACCATTC    ........T............................    GCGTCGGCCTCCAGCGAGTCGGGCAGCAGGAGAA
   2686035      37   100.0      35  AGCAGGAGAA    .....................................    TGGTGGATGTGGTGGTTCGACGCGTCCGGCACCCG
   2686107      37   100.0      35  CCGGCACCCG    .....................................    GTAATCATCCGCTGGCCGCCGCCCTTCCAGCGCAG
```

```
   2686179      37   100.0      32  TCCAGCGCAG    .....................................    CGCAACACGCCGATCCTCACGCTCGCGAAGAA
   2686248      37   100.0      36  TCGCGAAGAA    .....................................    GCTTCCTGCGAGACAACGACTCGGACCCGATCCCCA
   2686321      37   100.0      35  CCGATCCCCA    .....................................    CAGCTCCAGCGCACCGTCCGGTTATCGCTCCGGCG
   2686393      37   100.0      34  CGCTCCGGCG    .....................................    GCGGGCGGTCGATGAACACCTGATTGCCGAACCC
   2686464      37   100.0      34  TGCCGAACCC    .....................................    CGGTCTCCCTTCGCGGTCCGGAGGAGAAGGGGCC
   2686535      37   100.0      32  AGAAGGGGCC    .....................................    ATAGGGCCTGGGGAGCCATGGGCCCTTGAAGC
   2686604      37   100.0      32  CCCTTGAAGC    .....................................    ATAGGGCCTGGGGAGCCATGGGCCCTTGAAGC
   2686673      37   100.0      32  CCCTTGAAGC    .....................................    ATAGGGCCTGGGGAGCCATGGGCCCTTGAAGC
   2686742      37   100.0      32  CCCTTGAAGC    .....................................    ATAGGGCCTGGGGAGCCATGGGCCCTTGAAGC
   2686811      37   100.0      34  CCCTTGAAGC    .....................................    CGTGGTTTCGGGGCGGCGGCAGGTGGCTCCCGAC
   2686882      37   100.0      34  GGCTCCCGAC    .....................................    GCGCGATAGACGCTCATGGCCGTCTCGTCGTGGT
   2686953      37    97.3      34  TCGTCGTGGT    .....................T...............    GCGCGATAGACGCTCATGGCCGTCTCGTCGTGGT
   2687024      37    97.3      34  TCGTCGTGGT    .....................T...............    GCGCGATAGACGCTCATGGCCGTCTCGTCGTGGT
   2687095      37   100.0      35  TCGTCGTGGT    .....................................    ACATGGCTGACGGGCTTGCGCCGGACGCGGTTGTC
   2687167      37   100.0      32  CGCGGTTGTC    .....................................    ATAGGGCCTGGGGAGCCATGGGCCCTTGAAGC
   2687236      37   100.0      33  CCCTTGAAGC    .....................................    CGCCGACCGGGAGCGGGCCATGGAAATCCTGGT
   2687306      37   100.0      33  AAATCCTGGT    .....................................    CGCCGACCGGGAGCGGGCCATGGAAATCCTGGT
   2687376      37   100.0      33  AAATCCTGGT    .....................................    CGCCGACCGGGAGCGGGCCATGGAAATCCTGGT
   2687446      37   100.0      33  AAATCCTGGT    .....................................    CGCCGACCGGGAGCGGGCCATGGAAATCCTGGT
   2687516      37   100.0      33  AAATCCTGGT    .....................................    CGCCGACCGGGAGCGGGCCATGGAAATCCTGGT
   2687586      37    97.3      33  AAATCCTGGT    .....................T...............    GGCATCAGCAACGCCCGGTGCTCCTCGAATGCG
   2687656      37    94.6      36  CTCGAATGCG    ..........A.....................A....    GTCCTCTGCGCGTTTGACGTAGCCCAGCACCTTGCC
   2687729      37    94.6      36  GCACCTTGCC    .....................T.......A.......    ACCCGTGCCAGCGTCGAGGCCCGCCTGGGCGGGTAC
   2687802      37    97.3      36  GGGCGGGTAC    ................................A....    GTCCTCTGCGCGTTTGACGTAGCCCAGCACCTTGCC
   2687875      37   100.0          GCACCTTGCC    .....................................    CGAGTGAGCA
==========  ======  ======  ======  ==========    =====================================
        38      37              33                GTCGCTCCCCGTGAACGCGGGGAGCGTGGGTTGAAAC
```

#----- predicted by CRISPRFinder (the sets of identical spacers shown in red, green and blue are identical to each other)

```
# Program: Crispr Finder Program
# Author: Ibtissem GRISSA
# Rundate (GMT): 23/8/2013 1:43:22
# Report_file: /var/www/html/CRISPR/Server/.tmp/Output/139.80.26.223_Aug_21_2013_06_06_13/tmp_1/tmp_1_Crispr_4
########################################
#=======================================
#
# Sequence: tmp_1
# Description:
# Length: 9003593
# Id: NC_015711
#
#==========================================================================
# Crispr Rank in the sequence: 5
# Crispr_begin_position: 2682223      Crispr_end_position:  2687911
# DR: GTCGCTCCCCGTGAACGCGGGGAGCGTGGGTTGAAAC   DR_length: 37  Number_of_spacers: 79
#==========================================================================
```

Spacer_begin_position	Spacer_length	Spacer_sequence
2682260	38	GTCACCCACAGCCTGCCCAGCGGCGCCACCGGGCGCTT
2682335	73	ACCCTGGAGGTCGTAGGTCGAACTTCAAGCCGTTGAAACAGCGGGCCGAACATCGCCCGGCTGCCGGGCGCTT
2682445	34	AGCGGGCCGAACATCGCCCGGCTGCCGGGCGCTT
2682516	34	AAGGCGACCGTCCGCGCGCTGTCCACCACGGCCT
2682587	35	CCTCCTCCTGGAGGGCGGGGTGAATAGCCTGCGCG
2682659	32	GCAACATCCAGGTGAAGCCCGGCGCGGAAATC
2682728	33	TTCGAACTGCGCGACCGGCGCGTGCAGTTCACC
2682798	32	GCAACATCCAGGTGAAGCCCGGCGCGGAAATC
2682867	34	GCGTCGGCCTCCAGCGAGTCGGGCAGCAGGAGAA
2682938	35	TGGTGGATGTGGTGGTTCGACGCGTCCGGCACCCG
2683010	35	GTAATCATCCGCTGGCCGCCGCCCTTCCAGCGCAG
2683082	32	CGCAACACGCCGATCCTCACGCTCGCGAAGAA
2683151	34	GCGTCGGCCTCCAGCGAGTCGGGCAGCAGGAGAA
2683222	32	ACCGTCACCGAGGTTATCCCACCGCTCCCCAC
2683291	32	GCAACATCCAGGTGAAGCCCGGCGCGGAAATC
2683360	34	GCGGGAATCATTACCGACCCGCATGGTGTCCCGG
2683431	32	GCAACATCCAGGTGAAGCCCGGCGCGGAAATC
2683500	32	GCAACATCCAGGTGAAGCCCGGCGCGGAAATC
2683569	32	GCAACATCCAGGTGAAGCCCGGCGCGGAAATC
2683638	32	GCAACATCCAGGTGAAGCCCGGCGCGGAAATC
2683707	69	AAGATGACGACACCGCAGCGGGCGCACGTCTGGAAACGCAACATCCAGGTGAAGCCCGGCGCGGAAATC
2683813	36	CTTCCACCACTGCCAATGCGCCGCTGGGCACCATTC
2683886	34	GCGTCGGCCTCCAGCGAGTCGGGCAGCAGGAGAA
2683957	32	GCCATGCAGTGGCTGGAGGAGTTCGTCCTGCC

2684026	34	CCTCGCCACCCTCCGCCAAATTTGCCACCGCGTG
2684097	32	ACCGTGGACGGCCGCAACTGGCTGCCCTGCAC
2684166	35	AAGTCTTCCGTGTTCTCCATGTCGCTTCCCGCCTG
2684238	37	CCATGACGCTGCCCCTCTCGCGGGCCTCGAGCCCGAT
2684312	35	ACCACCTTGTCGCTTTGGTGGTCGGCGTAGTGGAT
2684384	32	GTCACCCACAGCCTGCCCAGCGGCGCCACCTG
2684453	33	ACCCTGGAGGTCGTAGGTCGAACTTCAAGCCGA
2684523	34	AAGGCGACCGTCCGCGCGCTGTCCACCACGGCCT
2684594	35	CCTCCTCCTGGAGGGCGGGGTGAATAGCCTGCGCG
2684666	33	TTCGAACTGCGCGACCGGCGCGTGCAGTTCACC
2684736	35	TGGCACTGCCGCTTGACCTCCGCAGGCTTGAAGGT
2684808	32	ACCGTCACCGAGGTTATCCCACCGCTCCCCAC
2684877	32	ACCGTCACCGAGGTTATCCCACCGCTCCCCAC
2684946	32	ACCGTCACCGAGGTTATCCCACCGCTCCCCAC
2685015	32	ACCGTCACCGAGGTTATCCCACCGCTCCCCAC
2685084	32	ACCGTCACCGAGGTTATCCCACCGCTCCCCAC
2685153	32	ACCGTCACCGAGGTTATCCCACCGCTCCCCAC
2685222	34	AAGGCGACCGTCCGCGCGCTGTCCACCACGGCCT
2685293	35	CCTCCTCCTGGAGGGCGGGGTGAATAGCCTGCGCG
2685365	33	TTCGAACTGCGCGACCGGCGCGTGCAGTTCACC
2685435	35	TGGCACTGCCGCTTGACCTCCGCAGGCTTGAAGGT
2685507	33	CCCTGACGCTGCCCCTCTCGCGGGCCTCGGGCC
2685577	34	AGCGGGCCGAACATCGCCCGGCTGCCGGGCGCTT
2685648	32	ACCGTCACCGAGGTTATCCCACCGCTCCCCAC
2685717	34	GCGGGAATCATTACCGACCCGCATGGTGTCCCGG

2685788	32	GCAACATCCAGGTGAAGCCCGGCGCGGAAATC
2685857	34	AAGATGACGACACCGCAGCGGGCGCACGTCTGCT
2685928	36	CTTCCACCACTGCCAATGCGCCGCTGGGCACCATTC
2686001	34	GCGTCGGCCTCCAGCGAGTCGGGCAGCAGGAGAA
2686072	35	TGGTGGATGTGGTGGTTCGACGCGTCCGGCACCCG
2686144	35	GTAATCATCCGCTGGCCGCCGCCCTTCCAGCGCAG
2686216	32	CGCAACACGCCGATCCTCACGCTCGCGAAGAA
2686285	36	GCTTCCTGCGAGACAACGACTCGGACCCGATCCCCA
2686358	35	CAGCTCCAGCGCACCGTCCGGTTATCGCTCCGGCG
2686430	34	GCGGGCGGTCGATGAACACCTGATTGCCGAACCC
2686501	34	CGGTCTCCCTTCGCGGTCCGGAGGAGAAGGGGCC
2686572	32	ATAGGGCCTGGGGAGCCATGGGCCCTTGAAGC
2686641	32	ATAGGGCCTGGGGAGCCATGGGCCCTTGAAGC
2686710	32	ATAGGGCCTGGGGAGCCATGGGCCCTTGAAGC
2686779	32	ATAGGGCCTGGGGAGCCATGGGCCCTTGAAGC
2686848	34	CGTGGTTTCGGGGCGGCGGCAGGTGGCTCCCGAC
2686919	34	GCGCGATAGACGCTCATGGCCGTCTCGTCGTGGT
2686990	34	GCGCGATAGACGCTCATGGCCGTCTCGTCGTGGT
2687061	34	GCGCGATAGACGCTCATGGCCGTCTCGTCGTGGT
2687132	35	ACATGGCTGACGGGCTTGCGCCGGACGCGGTTGTC
2687204	32	ATAGGGCCTGGGGAGCCATGGGCCCTTGAAGC
2687273	33	**CGCCGACCGGGAGCGGGCCATGGAAATCCTGGT**
2687343	33	**CGCCGACCGGGAGCGGGCCATGGAAATCCTGGT**
2687413	33	**CGCCGACCGGGAGCGGGCCATGGAAATCCTGGT**

2687483	33	**CGCCGACCGGGAGCGGGCCATGGAAATCCTGGT**
2687553	33	**CGCCGACCGGGAGCGGGCCATGGAAATCCTGGT**
2687623	33	GGCATCAGCAACGCCCGGTGCTCCTCGAATGCG
2687693	36	GTCCTCTGCGCGTTTGACGTAGCCCAGCACCTTGCC
2687766	36	ACCCGTGCCAGCGTCGAGGCCCGCCTGGGCGGGTAC
2687839	36	GTCCTCTGCGCGTTTGACGTAGCCCAGCACCTTGCC

#==
##

#-------- **predicted by CRISPRDetect** (partial output) (using default parameters)

Array 6 2680594-2687986 **** Predicted by CRISPRDetect 2.1 ***
>NC_015711|Myxococcus-fulvus HW-1 chromosome, complete genome. Array_Orientation: Forward

Position	Repeat	%id	Spacer	Repeat_Sequence	Spacer_Sequence	Insertion/Deletion
2680594	37	100.0	35	....	CTCTTGCAGATGATGCAGTGGGCGGTGGCGGGCTT	
2680666	37	100.0	33	....	TCTGGATGCGGAGCCGCTGGCATGACGTAGGCC	
2680736	37	100.0	35	....	CTCTTGCAGATGATGCAGTGGGCGGTGGCGGGCTT	
2680808	37	100.0	33	....	TCTGGATGCGGAGCCGCTGGCATGACGTAGGCC	
2680878	37	100.0	34	....	CGGTCGGCGTCCCACACGTAGTCGTGCCACCACC	
2680949	37	100.0	32	....	ACCGTCACCGAGGTTATCCCACCGCTCCCCAC	
2681018	37	100.0	34	....	GCGGGAATCATTACCGACCCGCATGGTGTCCCGG	
2681089	37	100.0	32	....	GCAACATCCAGGTGAAGCCCGGCGCGGAAATC	
2681158	37	100.0	34	....	AAGATGACGACACCGCAGCGGGCGCACGTCTGCT	
2681229	37	100.0	36	....	CTTCCACCACTGCCAATGCGCCGCTGGGCACCATTC	
2681302	37	97.3	34	.......T.............................	GCGTCGGCCTCCAGCGAGTCGGGCAGCAGGAGAA	
2681373	37	100.0	35	....	TGGTGGATGTGGTGGTTCGACGCGTCCGGCACCCG	
2681445	37	100.0	35	....	GTAATCATCCGCTGGCCGCCGCCCTTCCAGCGCAG	
2681517	37	100.0	32	....	CGCAACACGCCGATCCTCACGCTCGCGAAGAA	
2681586	37	100.0	36	....	GCTTCCTGCGAGACAACGACTCGGACCCGATCCCCA	
2681659	37	100.0	35	....	CAGCTCCAGCGCACCGTCCGGTTATCGCTCCGGCG	
2681731	37	100.0	37	....	GGTGGCGGCACCCGCCACCTGGGCGACGCTGGAAATG	
2681805	37	100.0	34	....	CGTGGTTTCGGGGCGGCGGCAGGTGGCTCCCGAC	
2681876	37	100.0	34	....	GCGCGATAGACGCTCATGGCCGTCTCGTCGTGGT	
2681947	37	100.0	34	....	GCGGGCGGTCGATGAACACCTGATTGCCGAACCC	
2682018	37	100.0	37	....	CCATGACGCTGCCCCTCTCGCGGGCCTCGAGCCCGAT	
2682092	37	100.0	22	....	ATCGCCCGGCTGCCGGGCGCTT	Deletion [2682151]
2682151	37	100.0	35	....	ACCACCTTGTCGCTTTGGTGGTCGGCGTAGTGGAT	
2682223	37	100.0	38	....	GTCACCCACAGCCTGCCCAGCGGCGCCACCGGGCGCTT	
2682298	37	100.0	73	....	ACCCTGGAGGTCGTAGGTCGAACTTCAAGCCGTTGAAACAGCGGGCCGAACATCGCCCGGCTGCCGGGCGCTT	
2682408	37	100.0	34	....	AGCGGGCCGAACATCGCCCGGCTGCCGGGCGCTT	
2682479	37	100.0	34	....	AAGGCGACCGTCCGCGCGCTGTCCACCACGGCCT	

2682550	37	100.0	35	....	CCTCCTCCTGGAGGGCGGGGTGAATAGCCTGCGCG
2682622	37	100.0	32	....	GCAACATCCAGGTGAAGCCCGGCGCGGAAATC
2682691	37	100.0	33	....	TTCGAACTGCGCGACCGGCGCGTGCAGTTCACC
2682761	37	100.0	32	....	GCAACATCCAGGTGAAGCCCGGCGCGGAAATC
2682830	37	100.0	34	....	GCGTCGGCCTCCAGCGAGTCGGGCAGCAGGAGAA
2682901	37	100.0	35	....	TGGTGGATGTGGTGGTTCGACGCGTCCGGCACCCG
2682973	37	100.0	35	....	GTAATCATCCGCTGGCCGCCGCCCTTCCAGCGCAG
2683045	37	100.0	32	....	CGCAACACGCCGATCCTCACGCTCGCGAAGAA
2683114	37	100.0	34	....	GCGTCGGCCTCCAGCGAGTCGGGCAGCAGGAGAA
2683185	37	100.0	32	....	ACCGTCACCGAGGTTATCCCACCGCTCCCCAC
2683254	37	100.0	32	....	GCAACATCCAGGTGAAGCCCGGCGCGGAAATC
2683323	37	100.0	34	....	GCGGGAATCATTACCGACCCGCATGGTGTCCCGG
2683394	37	100.0	32	....	GCAACATCCAGGTGAAGCCCGGCGCGGAAATC
2683463	37	100.0	32	....	GCAACATCCAGGTGAAGCCCGGCGCGGAAATC
2683532	37	100.0	32	....	GCAACATCCAGGTGAAGCCCGGCGCGGAAATC
2683601	37	100.0	32	....	GCAACATCCAGGTGAAGCCCGGCGCGGAAATC
2683670	37	100.0	69	....	AAGATGACGACACCGCAGCGGGCGCACGTCTGGAAACGCAACATCCAGGTGAAGCCCGGCGCGGAAATC
2683776	37	100.0	36	....	CTTCCACCACTGCCAATGCGCCGCTGGGCACCATTC
2683849	37	97.3	34	.......T.............................	GCGTCGGCCTCCAGCGAGTCGGGCAGCAGGAGAA
2683920	37	100.0	32	....	GCCATGCAGTGGCTGGAGGAGTTCGTCCTGCC
2683989	37	100.0	34	....	CCTCGCCACCCTCCGCCAAATTTGCCACCGCGTG
2684060	37	100.0	32	....	ACCGTGGACGGCCGCAACTGGCTGCCCTGCAC
2684129	37	100.0	35	....	AAGTCTTCCGTGTTCTCCATGTCGCTTCCCGCCTG
2684201	37	100.0	37	....	CCATGACGCTGCCCCTCTCGCGGGCCTCGAGCCCGAT
2684275	37	100.0	35	....	ACCACCTTGTCGCTTTGGTGGTCGGCGTAGTGGAT
2684347	37	100.0	32	....	GTCACCCACAGCCTGCCCAGCGGCGCCACCTG
2684416	37	100.0	33	....	ACCCTGGAGGTCGTAGGTCGAACTTCAAGCCGA
2684486	37	100.0	34	....	AAGGCGACCGTCCGCGCGCTGTCCACCACGGCCT
2684557	37	100.0	35	....	CCTCCTCCTGGAGGGCGGGGTGAATAGCCTGCGCG
2684629	37	100.0	33	....	TTCGAACTGCGCGACCGGCGCGTGCAGTTCACC
2684699	37	100.0	35	....	TGGCACTGCCGCTTGACCTCCGCAGGCTTGAAGGT
2684771	37	100.0	32	....	ACCGTCACCGAGGTTATCCCACCGCTCCCCAC
2684840	37	100.0	32	....	ACCGTCACCGAGGTTATCCCACCGCTCCCCAC
2684909	37	100.0	32	....	ACCGTCACCGAGGTTATCCCACCGCTCCCCAC
2684978	37	100.0	32	....	ACCGTCACCGAGGTTATCCCACCGCTCCCCAC
2685047	37	100.0	32	....	ACCGTCACCGAGGTTATCCCACCGCTCCCCAC
2685116	37	100.0	32	....	ACCGTCACCGAGGTTATCCCACCGCTCCCCAC
2685185	37	100.0	34	....	AAGGCGACCGTCCGCGCGCTGTCCACCACGGCCT
2685256	37	100.0	35	....	CCTCCTCCTGGAGGGCGGGGTGAATAGCCTGCGCG
2685328	37	100.0	33	....	TTCGAACTGCGCGACCGGCGCGTGCAGTTCACC
2685398	37	100.0	35	....	TGGCACTGCCGCTTGACCTCCGCAGGCTTGAAGGT
2685470	37	100.0	33	....	CCCTGACGCTGCCCCTCTCGCGGGCCTCGGGCC
2685540	37	100.0	34	....	AGCGGGCCGAACATCGCCCGGCTGCCGGGCGCTT
2685611	37	100.0	32	....	ACCGTCACCGAGGTTATCCCACCGCTCCCCAC
2685680	37	100.0	34	....	GCGGGAATCATTACCGACCCGCATGGTGTCCCGG
2685751	37	100.0	32	....	GCAACATCCAGGTGAAGCCCGGCGCGGAAATC
2685820	37	100.0	34	....	AAGATGACGACACCGCAGCGGGCGCACGTCTGCT
2685891	37	100.0	36	....	CTTCCACCACTGCCAATGCGCCGCTGGGCACCATTC
2685964	37	97.3	34	.......T.............................	GCGTCGGCCTCCAGCGAGTCGGGCAGCAGGAGAA
2686035	37	100.0	35	....	TGGTGGATGTGGTGGTTCGACGCGTCCGGCACCCG
2686107	37	100.0	35	....	GTAATCATCCGCTGGCCGCCGCCCTTCCAGCGCAG
2686179	37	100.0	32	....	CGCAACACGCCGATCCTCACGCTCGCGAAGAA
2686248	37	100.0	36	....	GCTTCCTGCGAGACAACGACTCGGACCCGATCCCCA
2686321	37	100.0	35	....	CAGCTCCAGCGCACCGTCCGGTTATCGCTCCGGCG
2686393	37	100.0	34	....	GCGGGCGGTCGATGAACACCTGATTGCCGAACCC
2686464	37	100.0	34	....	CGGTCTCCCTTCGCGGTCCGGAGGAGAAGGGGCC

```
   2686535          37   100.0      32  .....................................     ATAGGGCCTGGGGAGCCATGGGCCCTTGAAGC
   2686604          37   100.0      32  .....................................     ATAGGGCCTGGGGAGCCATGGGCCCTTGAAGC
   2686673          37   100.0      32  .....................................     ATAGGGCCTGGGGAGCCATGGGCCCTTGAAGC
   2686742          37   100.0      32  .....................................     ATAGGGCCTGGGGAGCCATGGGCCCTTGAAGC
   2686811          37   100.0      34  .....................................     CGTGGTTTCGGGGCGGCGGCAGGTGGCTCCCGAC
   2686882          37   100.0      34  .....................................     GCGCGATAGACGCTCATGGCCGTCTCGTCGTGGT
   2686953          37    97.3      34  .....................T...............     GCGCGATAGACGCTCATGGCCGTCTCGTCGTGGT
   2687024          37    97.3      34  .....................T...............     GCGCGATAGACGCTCATGGCCGTCTCGTCGTGGT
   2687095          37   100.0      35  .....................................     ACATGGCTGACGGGCTTGCGCCGGACGCGGTTGTC
   2687167          37   100.0      32  .....................................     ATAGGGCCTGGGGAGCCATGGGCCCTTGAAGC
   2687236          37   100.0      33  .....................................     CGCCGACCGGGAGCGGGCCATGGAAATCCTGGT
   2687306          37   100.0      33  .....................................     CGCCGACCGGGAGCGGGCCATGGAAATCCTGGT
   2687376          37   100.0      33  .....................................     CGCCGACCGGGAGCGGGCCATGGAAATCCTGGT
   2687446          37   100.0      33  .....................................     CGCCGACCGGGAGCGGGCCATGGAAATCCTGGT
   2687516          37   100.0      33  .....................................     CGCCGACCGGGAGCGGGCCATGGAAATCCTGGT
   2687586          37    97.3      33  .....................T...............     GGCATCAGCAACGCCCGGTGCTCCTCGAATGCG
   2687656          37    94.6      36  ..........A.....................A....     GTCCTCTGCGCGTTTGACGTAGCCCAGCACCTTGCC
   2687729          37    94.6      36  .....................T.......A.......     ACCCGTGCCAGCGTCGAGGCCCGCCTGGGCGGGTAC
   2687802          37    97.3      36  ................................A....     GTCCTCTGCGCGTTTGACGTAGCCCAGCACCTTGCC
   2687875          37   100.0      37  .....................................     CGAGTGAGCATTGCCACAGGTCTCTGTTTGTCAGTGA
   2687949          37    81.1       0  C..A..T.T......T...A....T............     |
==========       ======  ======  ======  =====================================     ========================================================================
      ==================
       104          37    99.5      34  GTCGCTCCCCGTGAACGCGGGGAGCGTGGGTTGAAAC

# Left flank :   AGGCGGTGGCATGGGAGGCCGGGTGGCTGCATGGCGCGGCCGGCAGGTTCGCGAAATGCGGCCGAATTCCGCAGGAAGATCGGTATGTTGGAAGGGCAGG
# Right flank :  GCATTAGCGAGCTTGAGTTGGCAGGCCGTTGGGGGAAGTCGCTCCTCGTGGCCGCTTGGCGCCGTATGGGCATCCAGTGGCCGTTTCTGGTGAGCGCACA

# Questionable array : NO  Score: 0.62
# Score Detail : 1:0, 2:0, 3:0, 4:0.97, 5:0, 6:1, 7:0.65, 8:-3, 9:1,
# Score Legend : 1: cas, 2: known_repeat, 3: motif_match, 4: overall_repeats_identity, 5: minimum_2_identical_repeats, 6: model_repeat_length, 7:spacer_lengths, 8:
spacer_identity, 9: log(no_of_perfect_repeats),
# Primary repeat :     GTCGCTCCCCGTGAACGCGGGGAGCGTGGGTTGAAAC
# Alternate repeat :   NA

# Directional analysis summary from each method:
#       Motif ATTGAAA(N) match prediction:         NA Score: 0/4.5
#       A,T distribution in repeat prediction:     NA [6,6] Score: 0.37/0.37
#       Reference repeat match prediction:         NA
#       Secondary Structural analysis prediction:  F [-12.10,-10.40] Score: 0.37/0.37
#       Array degeneracy analysis prediction:      F [1-14] Score: 0.41/0.41
#       AT richness analysis in flanks prediction: NA [40.0-35.0]%AT Score: 0/0.27
#       Longer leader analysis prediction:         NA [184,367] Score: 0/0.18
#       --------------------------------------------------------------------------
#       Final direction:         F [0.78,0   Confidence: HIGH]
```

S7C Example of array splitting due to non-identification of a propagating mutation in a CRISPR array. The CRISPR array from position 856227 to 857471 in the *Pyrobaculum neutrophilum* V24Sta chromosome contains mutation in the middle of the repeat (repeat no 12) that propagated to the next 6 repeats (up to repeat number 18). This event was not handled correctly by PILER-CR, which divided the array into two shorter ones with the following two representative repeats. As shown in the following alignment, both the

repeats are almost identical (except the bases shown in red) and should be predicted as a single array. CRISPRDetect uses methods like dynamic search to handle cases like this, which can adapt changes within an array. In the dynamic search method, instead of a fixed representative repeat, the closest repeat is used as the representative repeat during array extension. This ensures a better representation of the array as well as improves the array quality score.

```
Representative repeat1:      GAATCTCAAGTTGAGGATTGAAAG
                             ||||||||||  ||||||||||||
Representative repeat2:      GAATCTCAAAGAGAGGATTGAAAG
```

#------------------ predicted by PILER-CR

```
Array 4
>gi|171184485|ref|NC_010525.1|Pyrobaculumneutrophilum V24Sta chromosome, complete

       Pos  Repeat     %id  Spacer  Left flank    Repeat                      Spacer
==========  ======  ======  ======  ==========    ========================    ======
    856227      24   100.0      48  AGAAGACCCA    ........................    CGATCAGCTTGACGATCGTGGAGTGTATTACCGACTTCTGCTCCTCGG
    856299      24   100.0      45  TGCTCCTCGG    ........................    CGTAGTGGTAATCTCTAATCTCTCTAATGATGTCCTCATTCTCCA
    856368      24   100.0      42  TCATTCTCCA    ........................    CTTCATGACCGCATTAAATATATCGGGGTCTTGCATTGCTAC
    856434      24   100.0      41  GCATTGCTAC    ........................    CCTCGAGGAAGGCGTGGGGATCCCTGGCCAGCAGCTCAGCC
    856499      24   100.0      42  CAGCTCAGCC    ........................    TTTAACCGCAGAAACTTGTCGATAACTGAAAAAACGGGGTTG
    856565      24   100.0      45  AACGGGGTTG    ........................    TTGTACTCTTATAGAAACGTATTGTGGCCACCTTACGGCGGAGTG
    856634      24   100.0      44  CGGCGGAGTG    ........................    CAGTCTCCGCGGATGCTTGTGCATCGTTCGGCGCCGACAACTCA
    856702      24   100.0      41  CGACAACTCA    ........................    ATCTTCACAGCGTAGTACACCTGCGTGTGGCTGAGGGAGAG
    856767      24   100.0      38  TGAGGGAGAG    ........................    CGTCTAGGACGAGGGGCACTATCATTATGCGCCTGTCC
    856829      24   100.0      43  GCGCCTGTCC    ........................    CTCAGCTTGTAGACGTTCTCCATGTATTCATCGATATAGTACA
    856896      24   100.0          ATATAGTACA    ........................    CTCCACCA
==========  ======  ======  ======  ==========    ========================
        11      24              42                GAATCTCAAGTTGAGGATTGAAAG

Array 5
>gi|171184485|ref|NC_010525.1|Pyrobaculumneutrophilum V24Sta chromosome, complete

       Pos  Repeat     %id  Spacer  Left flank    Repeat                      Spacer
==========  ======  ======  ======  ==========    ========================    ======
    856966      24    95.8      48  CATGTATTGG    ..........A.............    CAAATACATGTAATACGTTGCAGTATTCTTATACAGCCTACTCTTTAC
    857038      24    95.8      43  TACTCTTTAC    ..........A.............    AATTGCCCCACGACTTGGGGGAGAGAAACGGCGGCGTGGGGGT
    857105      24   100.0      45  GCGTGGGGGT    ........................    AGGAGAGCGGCGTCGACGACGTCCGCCCTTCCGGGGAACTTGGGA
    857174      24    95.8      54  GAACTTGGGA    ..........A.............    ATAACATCCATAAGGTTTATTGGTCGTGCGAATGGCACCTCTTCATTGGGCAGA
    857252      24   100.0      41  ATTGGGCAGA    ........................    TCCACCACAGAGCCCGTAATTGTATACCACCGCGAATACCT
    857317      24   100.0      43  GCGAATACCT    ........................    ATCTGTATATGCGCCAACCTGTCAATAAGCGGGTCTGCGTTTT
    857384      24    95.8          TCTGCGTTTT    .......G................    TGCACCGGAA
==========  ======  ======  ======  ==========    ========================
```

```
        7      24               45                GAATCTCAAAGAGAGGATTGAAAG
```

#------------------- **Predicted by CRISPRDetect** (partial output) [array extension without using the Dynamic search method]

```
Array 5 856227-857472         **** Predicted by CRISPRDetect 2.1 ***
>gi|171184485|ref|NC_010525|-Pyrobaculum neutrophilum V24Sta chromosome, complete genome.        Array_Orientation: Forward

  Position     Repeat     %id  Spacer  Repeat_Sequence                Spacer_Sequence
       Insertion/Deletion
==========     ======  ======  ======  ========================       ======================================================
       ==================
    856227         24   100.0      48  ........................       CGATCAGCTTGACGATCGTGGAGTGTATTACCGACTTCTGCTCCTCGG
    856299         24   100.0      45  ........................       CGTAGTGGTAATCTCTAATCTCTCTAATGATGTCCTCATTCTCCA
    856368         24   100.0      42  ........................       CTTCATGACCGCATTAAATATATCGGGGTCTTGCATTGCTAC
    856434         24   100.0      41  ........................       CCTCGAGGAAGGCGTGGGGATCCCTGGCCAGCAGCTCAGCC
    856499         24   100.0      42  ........................       TTTAACCGCAGAAACTTGTCGATAACTGAAAAAACGGGGTTG
    856565         24   100.0      45  ........................       TTGTACTCTTATAGAAACGTATTGTGGCCACCTTACGGCGGAGTG
    856634         24   100.0      44  ........................       CAGTCTCCGCGGATGCTTGTGCATCGTTCGGCGCCGACAACTCA
    856702         24   100.0      41  ........................       ATCTTCACAGCGTAGTACACCTGCGTGTGGCTGAGGGAGAG
    856767         24   100.0      38  ........................       CGTCTAGGACGAGGGGCACTATCATTATGCGCCTGTCC
    856829         24   100.0      43  ........................       CTCAGCTTGTAGACGTTCTCCATGTATTCATCGATATAGTACA
    856896         24   100.0      46  ........................       CTCCACCAACGTCCTTATCTCTTTTAGGCATATTTCCATGTATTGG
    856966         24    87.5      48  .........AAA............       CAAATACATGTAATACGTTGCAGTATTCTTATACAGCCTACTCTTTAC
    857038         24    87.5      43  .........AAA............       AATTGCCCCACGACTTGGGGGAGAGAAACGGCGGCGTGGGGGT
    857105         23    91.7      45  ..........-A............       AGGAGAGCGGCGTCGACGACGTCCGCCCTTCCGGGGAACTTGGGA              A [857112]
    857174         24    87.5      54  .........AAA............       ATAACATCCATAAGGTTTATTGGTCGTGCGAATGGCACCTCTTCATTGGGCAGA
    857252         23    91.7      41  ..........-A............       TCCACCACAGAGCCCGTAATTGTATACCACCGCGAATACCT                  A [857259]
    857317         23    91.7      43  ..........-A............       ATCTGTATATGCGCCAACCTGTCAATAAGCGGGTCTGCGTTTT                A [857324]
    857384         23    91.7      40  ..........-A............       TGCACCGGAATGCACCGGAAAACCTACACTGTGCCCCTGA                   G [857391]
    857448         24    95.8       0  ......T.................       |
==========     ======  ======  ======  ========================       ======================================================
       ==================
        19         24    96.1      44  GAATCTCAAGTTGAGGATTGAAAG
```

#------- **Predicted by CRISPRDetect** [array extension using the Dynamic search method]

```
Array 5 856227-857471   **** Predicted by CRISPRDetect 2.1 ***
>gi|171184485|ref|NC_010525.1|Pyrobaculumneutrophilum V24Sta chromosome, complete genome   Array_Orientation: Unconfirmed

  Position     Repeat     %id  Spacer  Repeat_Sequence                Spacer_Sequence
       Insertion/Deletion
==========     ======  ======  ======  ========================       ======================================================
       ==================
    856227         24   100.0      48  ........................       CGATCAGCTTGACGATCGTGGAGTGTATTACCGACTTCTGCTCCTCGG
    856299         24   100.0      45  ........................       CGTAGTGGTAATCTCTAATCTCTCTAATGATGTCCTCATTCTCCA
```

```
    856368         24  100.0      42  ........................      CTTCATGACCGCATTAAATATATCGGGGTCTTGCATTGCTAC
    856434         24  100.0      41  ........................      CCTCGAGGAAGGCGTGGGGATCCCTGGCCAGCAGCTCAGCC
    856499         24  100.0      42  ........................      TTTAACCGCAGAAACTTGTCGATAACTGAAAAAACGGGGTTG
    856565         24  100.0      45  ........................      TTGTACTCTTATAGAAACGTATTGTGGCCACCTTACGGCGGAGTG
    856634         24  100.0      44  ........................      CAGTCTCCGCGGATGCTTGTGCATCGTTCGGCGCCGACAACTCA
    856702         24  100.0      41  ........................      ATCTTCACAGCGTAGTACACCTGCGTGTGGCTGAGGGAGAG
    856767         24  100.0      38  ........................      CGTCTAGGACGAGGGGCACTATCATTATGCGCCTGTCC
    856829         24  100.0      43  ........................      CTCAGCTTGTAGACGTTCTCCATGTATTCATCGATATAGTACA
    856896         24  100.0      46  ........................      CTCCACCAACGTCCTTATCTCTTTTAGGCATATTTCCATGTATTGG
    856966         24   87.5      48  .........AAA............      CAAATACATGTAATACGTTGCAGTATTCTTATACAGCCTACTCTTTAC
    857038         24   87.5      43  .........AAA............      AATTGCCCCACGACTTGGGGGAGAGAAACGGCGGCGTGGGGGT
    857105         24   87.5      45  .........AGA............      AGGAGAGCGGCGTCGACGACGTCCGCCCTTCCGGGGAACTTGGGA
    857174         24   87.5      54  .........AAA............      ATAACATCCATAAGGTTTATTGGTCGTGCGAATGGCACCTCTTCATTGGGCAGA
    857252         24   87.5      41  .........AGA............      TCCACCACAGAGCCCGTAATTGTATACCACCGCGAATACCT
    857317         24   87.5      43  .........AGA............      ATCTGTATATGCGCCAACCTGTCAATAAGCGGGTCTGCGTTTT
    857384         24   83.3      40  .......G.AGA............      TGCACCGGAATGCACCGGAAAACCTACACTGTGCCCCTGA
    857448         23   83.3       0  ......-T.AG.............      |                                                              T [857459]
==========     ====== ====== ======  ========================      ======================================================
       ==================
        19         24   94.3      44  GAATCTCAAGTTGAGGATTGAAAG
```

Additional File S8A Identification of tandem repeats. The region 982819 to 982966 in the *Bacillus cereus* ATCC 10987 complete genome contains 7 near identical 21 nt long repeats. This region was predicted as a CRISPR array by CRT but is not detected with a score > - 4.0 by CRISPRDetect. Note that the non-identical columns 3/4 matches at the beginning of the spacer make this appear like a CRISPR. CRISPRDetect has functions and scoring which eliminate such tandem repeats

#--------------------- predicted by CRT

```
>NC_003909
CRISPR 8   Range: 982851 - 983084
POSITION        REPEAT                          SPACER
--------        ------------------------        ----------------------------
982851          GTGGAGAAACAGAAACACCAGGTG        GAGAAACAGAAACACCAGGCGAAGAAACAGAGAAACCAG      [ 24, 39 ]
982914          GTGAAGAAACAGAAAAGCCAGGTG        AGGAAACAGAAAAGCCAGGCGAAGAGACAGGAAAACCGG      [ 24, 39 ]
982977          GTGAAGAAACAGAGAAACCAGGTG        AAGAGACAGGAAAACCGG     [ 24, 18 ]
983019          GTGAAGAAACAGAGAAGCCAGGTG        AAGAGACAGGAACATCAG     [ 24, 18 ]
983061          GTGAAGAAACAGAGAAGCCAGGTG
--------        ------------------------        ----------------------------
Repeats: 5      Average Length: 24              Average Length: 28
```

S8B Example of multiple identical spacers in an array. CRISPRs often contains multiple identical spacers together with non-identical spacers, as shown in the example CRISPR of *Methanocaldococcus jannaschii* DSM 2661 chromosome. The spacers in red are identical.

```
Array 4 236564-238185         **** Predicted by CRISPRDetect 2.1 ***
>gi|15668172|ref|NC_000909|-Methanocaldococcus jannaschii DSM 2661 chromosome, complete genome.          Array_Orientation: Forward

  Position     Repeat    %id  Spacer  Repeat_Sequence                        Spacer_Sequence
       Insertion/Deletion
==========     ======  ======  ======  ==============================        ==================================================
       ==================
    236564         30   100.0      42  ..............................        GATATTATTAAACAACATAATCAGTGTAATTGTATAGATATG
    236636         30   100.0      36  ..............................        ATTTGATGATTTGGTGGATTATACAAATAGAAATTA
    236702         30   100.0      39  ..............................        TACTGTTAAATATTCAGATTTATTAATCAGTTATTTCCT
    236771         30   100.0      38  ..............................        GATTTTCTTATGTTTAAAATCCTTATGAACGCTCGGAT
    236839         30   100.0      36  ..............................        TCTTTATCTCTCTTTACAGTATCGTATCTTAATTTT
    236905         30   100.0      51  ..............................        TTTTCAACAAGCATTTCTAACAAGTTTGGAGGTAATACTGCAACAATTTCA
    236986         30    93.3      38  G............................C        GTGATTGTAGAATTCTCATCTTCTTCTTGGGAGAGCCG
    237054         30   100.0      38  ..............................        GATTGGATGAGGGATATATCCAAAACTCAAAAGGATTG
    237122         30   100.0      37  ..............................        CTGTTAGGGAACCCTAAAAAGGTTCCCTTGAGGGTTC
    237189         30   100.0      37  ..............................        CTGTTAGGGAACCCTAAAAAGGTTCCCTTGAGGGTTC
    237256         30   100.0      37  ..............................        CTGTTAGGGAACCCTAAAAAGGTTCCCTTGAGGGTTC
    237323         30   100.0      37  ..............................        CTGTTAGGGAACCCTAAAAAGGTTCCCTTGAGGGTTC
    237390         30   100.0      37  ..............................        CTGTTAGGGAACCCTAAAAAGGTTCCCTTGAGGGTTC
    237457         30   100.0      37  ..............................        CTGTTAGGGAACCCTAAAAAGGTTCCCTTGAGGGTTC
```

```
   237524      30  100.0    37  ..............................     CTGTTAGGGAACCCTAAAAAGGTTCCCTTGAGGGTTC
   237591      30  100.0    43  ..............................     TCATTTGCATCATTTGTGCTGGGTCTGGCTGACTCTGTGTGTC
   237664      30   96.7    35  .............T................     TTCTTGGAATTGCTAAGTGGTTTATGCATAGTTGC
   237729      30  100.0    37  ..............................     ATGAGATTCATTCTTTGATCGAGGGCGATAGAGGTTC
   237796      30  100.0    39  ..............................     GAATTTTCGCACACGCGCTACATCTAATAAACAGATTTG
   237865      30  100.0    38  ..............................     GATGAAAAGAAAGCAATTGAAACAGCTATTATAACTTA
   237933      30  100.0    47  ..............................     ATACCATTAACAATTTCATATATTCTGTTTTTGTATTCAATCTTTTT
   238010      30   96.7    46  .............................G     CATAGATTATTTTTAAGCTGTTTTTTGGATTTTCTAATTTTAAATT      C [238039]
   238087      30   93.3    38  ..........................G..C     AATGTTCTAAATTCTCCTTGTAATTCTCCTAATGTTGT
   238155      30   96.7     0  ........................A.....     |
==========  ====== ====== ======  ==============================     ==================================================
      ==================
        24     30   99.0    39  ATTAAAATCAGACCGTTTCGGAATGGAAAT
```

Additional File S9 Schematic of removal of degenerated repeats at either end of CRISPRs. A. Before refinement. B. After refinement. The repeats with degeneracy above the cutoff are removed from either end, producing a CRISPR with higher quality score. This function is useful in predicting arrays, which could be otherwise excluded due to a poor quality score. Once the quality score is checked, and an array is identified to be a potential CRISPR, the CRISPRDetect pipeline will try to extend the arrays both ends with lower identity cutoff, to make the degenerated repeats be shown in the final output.

```
A.
                      Repeat                     Spacer
                      ========================  =====================
              ┌─────> A...C....T.........-----  ATATGCAA
              │       ........................  GGACAGCAACCCGTGTCGGAT
              │       ........................  TCACACGCGAATCGCCAATCG
 Degenerated          ........................  GATGTATGCCGACCGTGATTT
   repeats            ........................  AGATACGCCTTTACGTCGCCT
                      ........................  TAAAACACCGGTTGCGCAACC
              │       ........................  TCTAAATCTGTAACGTAGGAACC
              │       --..........A........---  AAATGCTAAGCTATTGCAGTATTCA
              └─────> .C..........T.....A..T..  GTACTGGTAC  -//-
                      ========================  =====================
                      CGGTTTATCCCCGCTGGCGCGGGG

B.
Repeat                     Spacer                 Insertion/Deletion
========================  =====================  ==================
........................  GGACAGCAACCCGTGTCGGAT
........................  TCACACGCGAATCGCCAATCG
........................  GATGTATGCCGACCGTGATTT
........................  AGATACGCCTTTACGTCGCCT
........................  TAAAACACCGGTTGCGCAACC
........................  TCTAAATCTGTAACGTAGGAA
.C..........A........---  AAATGC  -//-           Deletion  [X1]
========================  =====================  ==================
CGGTTTATCCCCGCTGGCGCGGGG
```

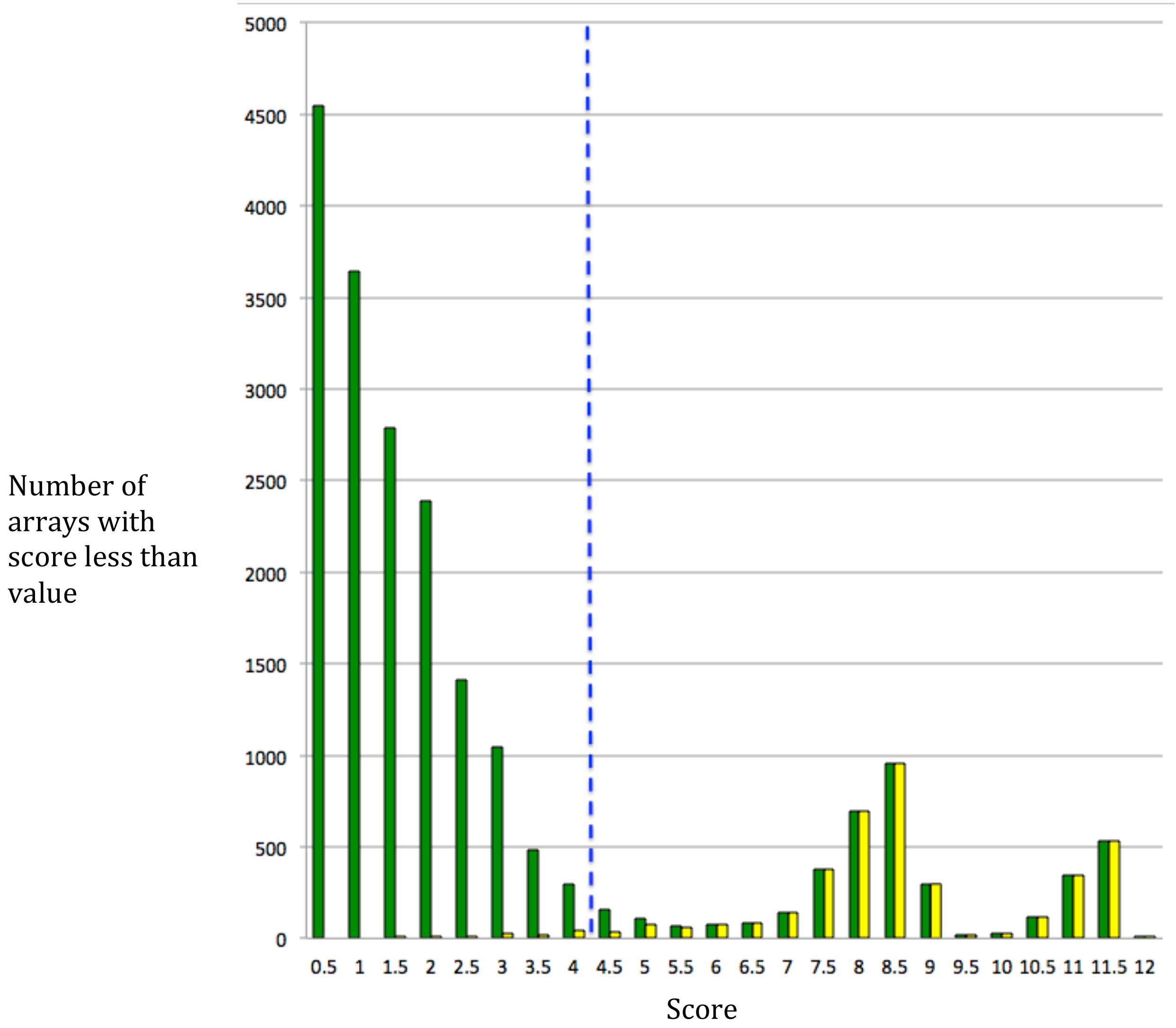

Additional file S10. Frequency counts for all predictions from CRISPRDetect. Only the scores above 0 are shown (these are included in CRISPRBank). Arrays with poor scores (<4.0 blue dotted line) would be flagged as 'questionable' in the output. Scores for CRISPRs with known repeats are shown as yellow bars, a match to these repeats adds additional +3 resulting in high scores.
